# US Cystic Fibrosis Foundation and European Cystic Fibrosis Society consensus recommendations for the management of non-tuberculous mycobacteria in individuals with cystic fibrosis

**DOI:** 10.1136/thoraxjnl-2015-207360

**Published:** 2015-12-11

**Authors:** R Andres Floto, Kenneth N Olivier, Lisa Saiman, Charles L Daley, Jean-Louis Herrmann, Jerry A Nick, Peadar G Noone, Diana Bilton, Paul Corris, Ronald L Gibson, Sarah E Hempstead, Karsten Koetz, Kathryn A Sabadosa, Isabelle Sermet-Gaudelus, Alan R Smyth, Jakko van Ingen, Richard J Wallace, Kevin L Winthrop, Bruce C Marshall, Charles S Haworth

**Affiliations:** 1 Cambridge Institute for Medical Research, University of Cambridge, Cambridge, UK; 2 Cambridge Centre for Lung Infection, Papworth Hospital, Cambridge, UK; 3 Cardiovascular and Pulmonary Branch, National Heart, Lung, and Blood Institute, NIH, Bethesda, Maryland, USA; 4 Department of Pediatrics, Columbia University Medical Center, Pediatric Infectious Diseases, New York, New York, USA; 5 Division of Mycobacterial and Respiratory Infections, National Jewish Health, Denver, Colorado, USA; 6 INSERM U1173, UFR Simone Veil, Versailles-Saint-Quentin University, Saint-Quentin en Yvelines, France; 7 AP-HP, Service de Microbiologie, Hôpital Raymond Poincaré, Garches, France; 8 Department of Medicine, National Jewish Health, Denver, Colorado, USA; 9 The University of North Carolina at Chapel Hill, Chapel Hill, North Carolina, USA; 10 Department of Respiratory Medicine, Royal Brompton Hospital, London, UK; 11 Department of Respiratory Medicine, Freeman Hospital, High Heaton, Newcastle, UK; 12 Department of Pediatrics University of Washington School of Medicine, Seattle, Washington, USA; 13 The Dartmouth Institute for Health Policy and Clinical Practice, Geisel School of Medicine at Dartmouth, Lebanon, New Hampshire, USA; 14 Department of Pediatrics, Sahlgrenska University Hospital, Gothenburg, Sweden; 15 Service de Pneumo-Pédiatrie, Université René Descartes, Hôpital Necker-Enfants Malades, Paris, France; 16 Division of Child Health, Obstetrics & Gynaecology, University of Nottingham, Nottingham, UK; 17 Department of Medical Microbiology, Radboud University Medical Center, Nijmegen, The Netherlands; 18 Department of Microbiology, University of Texas Health Science Center, Tyler, Texas, USA; 19 Oregon Health and Sciences University, Portland, Oregon, USA; 20 Cystic Fibrosis Foundation, Bethesda, Maryland, USA

**Keywords:** Cystic Fibrosis, Bacterial Infection

## Abstract

Non-tuberculous mycobacteria (NTM) are ubiquitous environmental organisms that can cause chronic pulmonary infection, particularly in individuals with pre-existing inflammatory lung disease such as cystic fibrosis (CF). Pulmonary disease caused by NTM has emerged as a major threat to the health of individuals with CF but remains difficult to diagnose and problematic to treat. In response to this challenge, the US Cystic Fibrosis Foundation (CFF) and the European Cystic Fibrosis Society (ECFS) convened an expert panel of specialists to develop consensus recommendations for the screening, investigation, diagnosis and management of NTM pulmonary disease in individuals with CF. Nineteen experts were invited to participate in the recommendation development process. Population, Intervention, Comparison, Outcome (PICO) methodology and systematic literature reviews were employed to inform draft recommendations. An anonymous voting process was used by the committee to reach consensus. All committee members were asked to rate each statement on a scale of: 0, completely disagree, to 9, completely agree; with 80% or more of scores between 7 and 9 being considered ‘good’ agreement. Additionally, the committee solicited feedback from the CF communities in the USA and Europe and considered the feedback in the development of the final recommendation statements. Three rounds of voting were conducted to achieve 80% consensus for each recommendation statement. Through this process, we have generated a series of pragmatic, evidence-based recommendations for the screening, investigation, diagnosis and treatment of NTM infection in individuals with CF as an initial step in optimising management for this challenging condition.

## Background

### Epidemiology of non-tuberculous mycobacteria in individuals with cystic fibrosis

Non-tuberculous mycobacteria (NTM) are increasingly being isolated from the sputum of adults and children with cystic fibrosis (CF), both in North America and in Europe.^1–17^ Estimates of the prevalence of NTM in the CF population have ranged from 1.3% in the earliest study reported in 1984^1^ to 32.7% in a review of individuals with CF over the age of 40 years in Colorado.^9^ To date, the largest studies published examined 986,^6^ 1216^15^ and 1582^17^ individuals with CF and reported rates of NTM-positive cultures of 13.0%, 13.7% and 6.6%, respectively. Recently, analysis of US Cystic Fibrosis Foundation (CFF) registry data has shown prevalence rates for NTM-positive culture in the USA of 12%^18^ but with considerable variation between individual states (0–28%).^19^

The NTM species most commonly identified in individuals with CF from North America and Europe are the slow-growing *Mycobacterium avium* complex (MAC) (including *M. avium*, *M. intracellulare* and *M. chimaera*), which can be found in up to 72% of NTM-positive sputum cultures,^6^ and the rapid-growing *M. abscessus* complex (MABSC) (comprising the subspecies *M. abscessus* subsp *abscessus* (*M. a. abscessus*), *M. a. bolletii*^20^ and *M. a. massiliense*^21^ ^22^ (the latter currently classified as part of *M. a. bolletii*)), which in many centres has now become the most common NTM isolated from individuals with CF.^7^ ^15^ ^17^ ^21^ ^23–25^ Other less commonly isolated species include *M. simiae*,^11^ *M. kansasii* and *M. fortuitum.*^26^ There are geographical differences in both the prevalence of NTM-positive cultures and also the relative frequency of different species seen between but also within countries.^6^ ^17^ ^19^ ^24^ ^25^ ^27^

NTM acquisition is strongly associated with age in individuals with CF, with prevalence increasing from 10% in children aged 10 years, to over 30% in adults over the age of 40 years.^9^ In individuals with an adult diagnosis of CF, over 50% (mostly females) have NTM-positive airway cultures.^9^ There appear to be species-specific differences in age-related prevalence within CF cohorts, with MAC more commonly isolated from adults over 25 years of age,^6^ ^7^ ^14^ ^17^ ^27^ while MABSC is isolated from all age groups, but peaks between those 11 and 15 years of age in some studies.^17^ ^28^ There may also be species-specific differences in virulence: individuals with MABSC-positive cultures are more likely to meet American Thoracic Society (ATS)/Infectious Diseases Society of America (IDSA) criteria for diagnosing NTM pulmonary disease (NTM-PD, see Diagnosis of NTM-PD in CF section), and have worse morbidity and mortality associated with a more rapid decline in lung function.^15^ ^27^ ^29^ ^30^

There has been a rise over the last four decades in the reported prevalence of NTM-positive cultures in respiratory samples from individuals with CF,^1^ ^6^ ^15^ ^17^ ^18^ ^23^ an increase in part mirroring temporal changes seen in the non-CF cohort.^31–38^ While increasing detection rates may reflect enhanced surveillance and/or improved microbiological detection,^6^ ^27^ ^39–42^ there are a number of lines of evidence suggesting a true rise in the frequency of NTM infection. A number of CF studies^43^ show year on year increases in NTM-positive cultures with no change in surveillance intensity or culture methodology. There has been an increase over time in rates of skin test reactivity to NTM antigens in US population-based testing studies,^44^ potentially indicating increasing exposure to NTM (see below). Furthermore, the relative frequency of *M. abscessus* detection in NTM-positive samples from individuals with CF has increased remarkably over time both in the USA and in Europe,^2^ ^6^ ^15^ ^17^ ^23^ ^27^ suggesting real changes in NTM acquisition rates (rather than increased sampling).

Possible reasons for the potential increased frequency of NTM-positive cultures in individuals with CF include: increases in environmental exposure to NTM through more permissive temperature settings of home water heaters^45^ and more contact with shower aerosols,^46^ ^47^ increased antibiotic usage creating more NTM favourable lung niches,^27^ greater chronic use of medications that might impair host immunity to NTM,^43^ and/or spread of NTM through person-to-person transmission.^48^ ^49^

#### NTM-PD in individuals with CF

NTM can cause progressive inflammatory lung damage, a condition termed ‘NTM pulmonary disease’ (NTM-PD),^50^ ^51^ which is defined by the presence of specific microbiological, clinical and radiological features described in Diagnosis of NTM-PD in CF section. However, it has become clear that NTM can also transiently, intermittently or permanently reside within the lungs of individuals with CF without causing NTM-PD, thus representing asymptomatic infection and creating considerable difficulties in deciding how best to screen for and diagnose NTM.^30^ Further challenges exist in knowing how best to identify NTM in respiratory samples, when and how to initiate treatment for NTM-PD (as highlighted by a recent Cochrane review^52^) and how NTM may impact individuals under consideration for lung transplantation. As a consequence, the CFF and European Cystic Fibrosis Society (ECFS) sought to generate a consensus recommendations document to support and standardise the management of NTM infection in individuals with CF, permitting prospective evaluation of current best practice and forming a foundation for future research programmes.

These consensus statements have been developed to assist in the management both of adults and children with CF who are infected with NTM. Given the virtual absence of published evidence to guide paediatric care,^53^ recommendations for children with CF infected with NTM are based on extrapolated adult data, the practical experience of experts and appropriate adjustment of drug regimens, and are, except where stated, the same as for adults.

## Methods

### Expert committee structure

The CFF and the ECFS invited experts to participate in the statement development process. The 19-member committee consisted of professionals (10 US and 9 European) with expertise in CF and NTM, and included adult and paediatric CF physicians, lung transplant physicians, microbiologists, infectious disease specialists and a parent of an individual with CF. The committee convened in May 2012 and was divided into five subgroups, each responsible for a specific topic: Epidemiology and Risk Factors, Screening, Microbiology, Treatment and Transplantation. Each subgroup developed topic-specific questions using the PICO format (Population, Intervention, Comparison, Outcome.^54^) Questions were reviewed and approved by the entire committee before systematic literature searches were conducted.

### Review process and consensus vote

The members of each subgroup used the PICO questions to guide literature searches in PubMed. Searches were limited to the English language and the period 1984 to 2013. Subgroup members also searched for topic-relevant guidelines through searches of the ATS website, the IDSA website, the Clinical Laboratory Standards Institute (CLSI) website and the UK CF Trust website.

After reviewing the relevant literature and existing guidelines, subgroup members drafted recommendation statements. In October 2012, a second meeting was convened and subgroups finalised draft recommendation statements. The committee also voted to set 80% agreement of all 19 members as the threshold for acceptance of a recommendation statement and not to use the GRADE system of evaluating published evidence, given the paucity of clinical trial data.

Each subgroup submitted final draft questions for entry into an electronic survey tool (Survey Monkey) for the purposes of anonymous voting and comment by all members. A project coordinator administered the survey and committee members were asked to rate each statement on a scale of: 0, completely disagree, to 9, completely agree; with 80% or between 7 and 9 being considered ‘good’ agreement. Space for entering free text was also provided after each statement to allow members to cite literature in support of their opinions or suggested revisions. All committee members were required to vote on each statement regardless of their role or expertise. Multiple rounds of voting and revisions to the statements were conducted, and for each round committee members were requested to complete their voting within 3 weeks. The committee chairs reviewed the results from each round and updated the statements based on comments entered by respondents for subsequent rounds.

### External review

A draft of the recommendations was presented at the 2013 North American Cystic Fibrosis Conference and the European Cystic Fibrosis Society Meeting. Additionally, the committee solicited feedback from the CF communities in the USA and in Europe, which included physicians, nurses, physical and respiratory therapists, parents and individuals with CF. All comments collected from this process were reviewed and addressed by the committee in the development of the final recommendation statements.

## Results

### Final recommendations and results of the consensus vote

Three rounds of voting were conducted to achieve 80% consensus for each statement. Fifty-three statements were included in the first round of voting and 50 statements in the second and third rounds. Final statements and the consensus are reported in table 1.

**Table 1:** NTM recommendation statements

Recommendation	Consensus (%)
*Recommendation 1*: The CF Foundation and the ECFS recommend that the potential for cross-infection of NTM (particularly *Mycobacterium abscessus* complex) between individuals with CF should be minimised by following national infection control guidelines	94
*Recommendation 2*: The CF Foundation and the ECFS recommend that cultures for NTM be performed annually in spontaneously expectorating individuals with a stable clinical course	94
*Recommendation 3*: The CF Foundation and the ECFS recommend that, in the absence of clinical features suggestive of NTM pulmonary disease, individuals who are not capable of spontaneously producing sputum do not require screening cultures for NTM	100
*Recommendation 4*: The CF Foundation and the ECFS recommend that culture and smears for AFB from sputum should be used for NTM screening	100
*Recommendation 5*: The CF Foundation and the ECFS recommend against the use of oropharyngeal swabs for NTM screening	100
*Recommendation 6*: The CF Foundation and the ECFS recommend that culture and smears for AFB from sputum, induced sputum, bronchial washings or bronchoalveolar lavage samples can be used to evaluate individuals with CF suspected to have NTM pulmonary disease.	100
*Recommendation 7*: The CF Foundation and the ECFS recommend against the routine use of transbronchial biopsies to detect NTM in individuals with CF suspected to have NTM pulmonary disease	100
*Recommendation 8*: The CF Foundation and the ECFS recommend against the use of oropharyngeal swabs to perform diagnostic smears and cultures in individuals with CF suspected to have NTM pulmonary disease	100
*Recommendation 9*: The CF Foundation and the ECFS recommend that respiratory tract samples should be cultured using both solid and liquid media	100
*Recommendation 10*: The CF Foundation and the ECFS recommend that the incubation duration for NTM cultures should be for a minimum of 6 weeks	100
*Recommendation 11*: The CF Foundation and the ECFS recommend that an NTM culture should be processed within 24 h of collection to optimise the detection of NTM in respiratory samples. If a delay in processing is anticipated, refrigeration of samples is advised	100
*Recommendation 12*: The CF Foundation and the ECFS recommend that respiratory tract samples should be decontaminated using the standard *N*-acetyl l-cysteine, NALC, (0.5%)–NaOH (2%) method	100
*Recommendation 13*: The CF Foundation and the ECFS recommend that, if a sample remains contaminated with Gram-negative bacteria after standard NALC-NaOH decontamination, it should be further treated with either 5% oxalic acid or 1% chlorhexidine	100
*Recommendation 14*: The CF Foundation and the ECFS recommend against the use of non-culture-based methods for detecting NTM in respiratory tract samples	100
*Recommendation 15*: The CF Foundation and the ECFS recommend that all NTM isolates from individuals with CF should undergo molecular identification	100
*Recommendation 16*: The CF Foundation and the ECFS recommend that all NTM isolates from individuals with CF should be identified to the species level, except for *M. intracellulare, M. avium and M. chimaera*, where identification can be limited to MAC, and *M. abscessus* complex, which should be subspeciated	83
*Recommendation 17*: The CF Foundation and the ECFS recommend that for MAC, clarithromycin susceptibility testing should be performed on an isolate recovered prior to initiation of treatment. Clarithromycin susceptibility testing should also be performed on subsequent isolates if the patient (a) fails to culture convert after 6 months of NTM treatment; (b) recultures MAC after initial culture conversion while on NTM treatment or (c) recultures MAC after completion of NTM treatment	94
*Recommendation 18*: The CF Foundation and the ECFS recommend that for *M. abscessus* complex, susceptibility testing should include at least clarithromycin, cefoxitin and amikacin (and preferably also tigecycline, imipenem, minocycline, moxifloxacin and linezolid)	89
*Recommendation 19*: The CF Foundation and the ECFS recommend that drug susceptibility testing should be performed in accordance with CLSI guidelines	100
*Recommendation 20*: The CF Foundation and the ECFS recommend that ATS/IDSA criteria for the diagnosis of NTM pulmonary disease should be used in individuals with CF (ATS/IDSA 2007 Statement)	100
*Recommendation 21*: The CF Foundation and the ECFS recommend that other CF pathogens and comorbidities should be considered as potential contributors to a patient's symptoms and radiological features when determining the clinical significance of NTM-positive cultures	100
*Recommendation 22*: The CF Foundation and the ECFS recommend that NTM treatment should be considered for individuals with CF who have ATS/IDSA defined NTM pulmonary disease	100
*Recommendation 23*: The CF Foundation and the ECFS recommend that individuals receiving azithromycin as part of their CF medical regimen who have a positive NTM culture should not continue azithromycin treatment while evaluation for NTM disease is underway as azithromycin monotherapy may lead to resistance. A macrolide agent may be included in a multidrug treatment regimen if criteria are met for NTM disease	89
*Recommendation 24*: The CF Foundation and the ECFS recommend that treatment of *M. abscessus* complex pulmonary disease should involve an intensive phase followed by a continuation phase	100
*Recommendation 25*: The CF Foundation and the ECFS recommend that the intensive phase should include a daily oral macrolide (preferably azithromycin) in conjunction with 3–12 weeks of intravenous amikacin and one or more of the following: intravenous tigecycline, imipenem or cefoxitin, guided but not dictated by drug susceptibility testing. The duration of intensive phase therapy should be determined by the severity of infection, the response to treatment and the tolerability of the regimen	83
*Recommendation 26*: The CF Foundation and the ECFS recommend that the continuation phase should include a daily oral macrolide (preferably azithromycin) and inhaled amikacin, in conjunction with 2–3 of the following additional oral antibiotics: minocycline, clofazimine, moxifloxacin and linezolid, guided but not dictated by drug susceptibility testing	89
*Recommendation 27*: The CF Foundation and the ECFS recommend that individuals with *M. abscessus* complex pulmonary disease should be managed in collaboration with experts in the treatment of NTM and CF, as drug intolerance and drug-related toxicity occur frequently, and changes in antibiotic therapy are often required	89
*Recommendation 28*: The CF Foundation and the ECFS recommend that monotherapy with a macrolide or other antimicrobial should never be used in the treatment of *M. abscessus* complex pulmonary disease	100
*Recommendation 29*: The CF Foundation and the ECFS recommend the same antibiotic regimen for treatment of all species within the MAC	94
*Recommendation 30*: The CF Foundation and the ECFS recommend that clarithromycin-sensitive MAC pulmonary disease should be treated with a daily oral antibiotic regimen containing a macrolide (preferably azithromycin), rifampin and ethambutol	89
*Recommendation 31*: The CF Foundation and the ECFS recommend against the use of intermittent (three times per week) oral antibiotic therapy to treat MAC pulmonary disease	89
*Recommendation 32*: The CF Foundation and the ECFS recommend that monotherapy with a macrolide or other antimicrobial agent should never be used in the treatment of MAC pulmonary disease	100
*Recommendation 33*: The CF Foundation and the ECFS recommend that an initial course of intravenous amikacin should be considered for the treatment of MAC pulmonary disease in the presence of one or more of the following: AFB smear positive respiratory tract samples Radiological evidence of lung cavitation or severe infection Systemic signs of illness	94
*Recommendation 34*: The CF Foundation and the ECFS recommend that clarithromycin-resistant MAC pulmonary disease should be managed in collaboration with experts in the treatment of NTM and CF	89
*Recommendation 35*: The CF Foundation and the ECFS recommend that individuals with CF receiving NTM treatment should have expectorated or induced sputum samples sent for NTM culture every 4–8 weeks throughout the entire course of treatment to assess the microbiological response	94
*Recommendation 36*: The CF Foundation and the ECFS recommend that a schedule for detecting drug toxicity (including hearing loss, visual loss, renal impairment and liver function test abnormalities) should be set in place at the time of NTM treatment initiation and implemented throughout treatment based on the specific drugs prescribed	100
*Recommendation 37*: The CF Foundation and the ECFS recommend that an HRCT scan of the lungs should be performed shortly before starting NTM treatment and at the end of NTM treatment to assess the radiological response	94
*Recommendation 38*: The CF Foundation and the ECFS recommend that NTM antibiotic therapy should be prescribed for 12 months beyond culture conversion (defined as three consecutive negative cultures, with the time of conversion being the date of the first of the three negative cultures) as long as no positive cultures are obtained during those 12 months	94
*Recommendation 39*: The CF Foundation and the ECFS recommend that individuals who fail to culture convert despite optimal NTM therapy may benefit from long-term suppressive antibiotic treatment	94
*Recommendation 40*: The CF Foundation and the ECFS recommend that, when amikacin is given intravenously or when streptomycin is given intravenously or intramuscularly, serum levels should be monitored and dosing adjusted to minimise ototoxicity and nephrotoxicity	100
*Recommendation 41*: The CF Foundation and the ECFS recommend against routinely obtaining serum levels of other anti-mycobacterial drugs. However, absorption of oral medications is often reduced in CF. Therefore use of therapeutic drug monitoring should be considered for individuals failing to improve despite taking recommended drug regimens or for those on concomitant medications with significant interactions with NTM drugs	100
*Recommendation 42*: The CF Foundation and the ECFS recommend against the use of interferon γ as adjuvant therapy for NTM pulmonary disease in individuals with CF	89
*Recommendation 43*: The CF Foundation and the ECFS recommend that vitamin D should be supplemented according to national CF care guidelines	94
*Recommendation 44*: The CF Foundation and the ECFS recommend that lung resection should only be considered under extraordinary circumstances and in consultation with experts on the treatment of NTM and CF	83
*Recommendation 45*: The CF Foundation and the ECFS recommend that all individuals with CF being considered for lung transplantation should be evaluated for NTM pulmonary disease	100
*Recommendation 46*: The CF Foundation and the ECFS recommend that the presence of current or previous respiratory tract samples positive for NTM should not preclude individuals being considered for lung transplantation	94
*Recommendation 47*: The CF Foundation and the ECFS recommend that individuals with CF who have NTM pulmonary disease and are being evaluated for transplantation should start treatment prior to transplant listing	100
*Recommendation 48*: The CF Foundation and the ECFS recommend that individuals with CF receiving NTM treatment with sequential negative cultures may be eligible for transplant listing	100
*Recommendation 49*: The CF Foundation and the ECFS recommend that individuals with CF who have completed treatment for NTM pulmonary disease with apparent eradication of the organism may be eligible for transplant listing	100
*Recommendation 50*: The CF Foundation and the ECFS recommend that the presence of persistent *M. abscessus* complex or MAC infection despite optimal therapy is not an absolute contraindication to lung transplant referral	94

AFB, acid-fast bacilli; CF, cystic fibrosis; CLSI, Clinical Laboratory Standards Institute; ECFS, European Cystic Fibrosis Society; HRCT, High-resolution CT; MAC, *M. avium* complex; NTM, non-tuberculous mycobacteria.

## Risk factors

### Are there modifiable risk factors for the development of NTM-PD in individuals with CF?

***Recommendation 1*: The CF Foundation and the ECFS recommend that the potential for cross-infection of NTM (particularly MABSC) between individuals with CF should be minimised by following national infection control guidelines.** CF-related lung disease is a clear risk factor for the development of NTM-PD and is presumed to relate to the presence of structural lung damage, impaired mucociliary clearance and inflamed airways; all of which are thought to favour the development of chronic NTM infection.^55^ Cystic Fibrosis Transmembrane conductance Regulator (CFTR) dysfunction may, of itself, predispose to NTM infection (although the pathophysiology is unknown), since rates of heterozygosity for CFTR mutations within the non-CF population with pulmonary NTM disease are high (30–50%).^56^ ^57^

However, other risk factors that predispose specific individuals with CF to acquire NTM or to develop NTM-PD are, for the most part, poorly understood, with many studies presenting conflicting results. Potential risk factors for NTM acquisition are listed below.

#### Lung function

There have been conflicting reports on whether an individual's spirometry results are related to the likelihood of finding NTM-positive samples, with some studies suggesting no association with lung function,^13^ a positive association of NTM acquisition with higher FEV_1_% predicted^6^ or, conversely, with worse lung function.^11^ ^15^ ^30^ Support for the possibility that NTM acquisition is more likely in CF individuals with severe lung disease comes from observations that the prevalence of NTM-positive sputum samples in patients referred for lung transplantation has been reported to be as high as 19.7%.^29^

#### Lung infection with specific pathogens

In some studies, individuals with CF with NTM-positive samples are more likely to have *Staphylococcus aureus* infection and less likely to have *Pseudomonas aeruginosa* chronic pulmonary infection.^6^ ^7^ ^58^ Other studies, however, have reported NTM positivity associated with higher rates of *P. aeruginosa* infection,^11^ and variably associated with *S. maltophilia* infection.^6^ ^58^ In contrast, *Aspergillus fumigatus* has consistently been associated with the presence of NTM-positive cultures,^11^ ^15^ ^59^ with some reports indicating an association with allergic bronchopulmonary aspergillosis.^7^ ^27^ ^60^

#### Medications

##### Corticosteroids

The impact of systemic steroids on NTM acquisition is controversial. There have been suggestions that steroids may protect against^58^ or predispose towards NTM infection,^60^ or may not influence the risk of NTM acquisition.^4^ ^11^ ^12^ Recent data from non-CF populations, however, have suggested that oral as well as some types of inhaled corticosteroids are associated with increased risk of NTM acquisition.^61–63^

##### Proton pump inhibitors

The impact of proton pump inhibitor (PPI) is unclear. PPI use has been reported to be associated with the development of MAC pulmonary disease in non-CF cohorts,^64^ and may promote gastrointestinal survival of NTM and subsequent lung infection through gastric aspiration.

##### Azithromycin

Particular attention has recently been paid to the role of long-term azithromycin use as a risk factor for the acquisition of NTM. In a single centre study of CF adults, Renna *et al*^43^ reported increases in annual rates of NTM infection associated with chronic azithromycin use, postulating, through in vitro studies and mouse infection models, that azithromycin blocked autophagic killing of NTM within macrophages. While supporting findings from a previous case–control study reporting increased azithromycin use in individuals with NTM,^11^ other large retrospective studies have shown no such association.^12^ ^13^ ^59^ ^65–67^ This includes a recent nested case–control analysis within the CF registry, which suggested long-term azithromycin use may protect against infection with NTM.^67^

#### Acquisition of NTM through cross-infection

Person-to-person transmission of NTM has traditionally been considered unlikely. Two separate studies have shown that patients, even siblings living in the same household for more than 10 years, have unique strains,^7^ ^68^ suggesting a lack of person-to-person transmission. However, a case report from the University of Washington described a possible outbreak of *M. a. massiliense* in five patients^48^ with potential transmission occurring during synchronous clinic visits. Recently, whole genome sequencing and antimicrobial susceptibility testing performed on 168 consecutive isolates of *M. abscessus* from 31 patients attending an adult CF centre in the UK revealed frequent, probably indirect, transmission of *M. a. massiliense* between individuals with CF despite conventional cross-infection measures.^69^ The results of these studies indicate that cross-infection may be an important mechanism for the acquisition of *M. abscessus* (at least within the CF population). To date, there has been no published evidence suggesting person-to-person transmission of other NTM species.

Other factors extrapolated from data in non-CF populations or studies on *M. tuberculosis* that might contribute to NTM acquisition in individuals with CF include: low vitamin D,^70^ ^71^ the presence of gastro-oesophageal reflux disease,^64^ ^72^ low body mass index^56^ ^73^ or malnutrition.^74^

## Screening

### How often should individuals with CF be screened for NTM?

***Recommendation 2*: The CF Foundation and the ECFS recommend that cultures for NTM be performed annually in spontaneously expectorating individuals with a stable clinical course.**

***Recommendation 3*: The CF Foundation and the ECFS recommend that, in the absence of clinical features suggestive of NTM-PD, individuals who are not capable of spontaneously producing sputum do not require screening cultures for NTM.**

Over the past two decades, a number of expert opinions and reviews have urged routine screening for NTM in the general CF population. However, the optimal frequency and methodology for NTM surveillance in individuals with CF are not known. NTM are common in the environment, and are likely to be transiently introduced on a regular basis into the airways of individuals with CF. More frequent screening will, therefore, result in detection of more positive cultures,^11^ many of which will not be associated with the presence of NTM-PD,^6^ ^30^ ^58^ generating anxiety in patients and caregivers and initiating further (potentially invasive) investigations. However, signs and symptoms of NTM disease are often subtle and non-specific, and the diagnosis can be delayed for years or missed altogether in the absence of effective surveillance.^4^ Furthermore, systematic screening may help researchers more accurately identify factors influencing poorly understood host susceptibility, acquisition, transmission and virulence of NTM. It is important to emphasise that screening refers to obtaining samples from individuals with no clinical, microbiological or radiological suspicion of NTM infection, and should be distinguished from strategies to investigate and diagnose NTM disease (covered in Diagnosis of NTM-PD in CF section).

While our understanding of those factors predisposing individuals with CF to NTM infection is incomplete, there is, nevertheless, agreement that certain patient populations are at greater risk and therefore probably require more frequent surveillance. These populations include: those with advanced lung disease and previous NTM-positive cultures, and those living in areas with high NTM prevalence. Conversely, in individuals with no recognised risk factors, the prevalence of NTM infection is likely to be low; thus less frequent, perhaps annual, surveillance is warranted. In addition, NTM screening is important before starting long-term azithromycin treatment to avoid inadvertent macrolide monotherapy in individuals with undiagnosed NTM infection (in keeping with published guidelines.^75^)

### How should screening for NTM be performed?

***Recommendation 4*: The CF Foundation and the ECFS recommend that culture and smears for acid-fast bacilli (AFB) from sputum should be used for NTM screening.**

***Recommendation 5*: The CF Foundation and the ECFS recommend against the use of oropharyngeal swabs for NTM screening.**

The majority of published reports describing the prevalence of NTM in the CF population utilised AFB smear and culture from sputum as the standard screening method.^4^ ^6^ ^7^ ^11^ ^13^ ^17^ To date, there has been no direct comparison between the sensitivity of samples from spontaneously expectorated sputum samples, and sputum induced by use of hypertonic saline. Analysis of induced sputum provides equal or better detection of ‘standard’ CF pathogens^76^ and the procedure is in widespread use to collect samples for mycobacterial culture among CF Centres worldwide. However, the Consensus Committee felt that, due to its inconvenience, induced sputum collection should not be used as a screening tool in individuals with no features suggestive of NTM-PD who are incapable of spontaneously producing sputum. As discussed in Microbiology section, there are currently no other validated screening methods to detect NTM in individuals with CF. Although positive cultures have been detected through laryngeal suction, oropharyngeal swabs, or gastric aspirate, there are insufficient data to support their use. Skin testing for delayed-type hypersensitivity against NTM antigens does not appear sufficiently sensitive or specific to use for surveillance in the CF population. Serological assays, such as IgG against *Mycobacterium* antigen A60 for NTM surveillance, appear promising,^42^ but have not been validated in the CF population.

## Microbiology

### What respiratory tract samples should be used to evaluate individuals with CF for suspected NTM-PD?

***Recommendation 6*: The CF Foundation and the ECFS recommend that culture and smears for AFB from sputum, induced sputum, bronchial washings or bronchoalveolar lavage samples can be used to evaluate individuals with CF suspected to have NTM-PD.**

***Recommendation 7*: The CF Foundation and the ECFS recommend against the routine use of transbronchial biopsies to detect NTM in individuals with CF suspected to have NTM-PD.**

***Recommendation 8*: The CF Foundation and the ECFS recommend against the use of oropharyngeal swabs to perform diagnostic smears and cultures in individuals with CF suspected to have NTM-PD.**

Currently, sputum, induced sputum, bronchial washings and bronchoalveolar lavage samples are routinely used to evaluate individuals for suspected NTM-PD.^77^ Samples for NTM should be processed for smear microscopy, preferably by fluorescence, and for culture. Microscopy allows for direct evaluation of the bacterial burden, and may indicate false-negative culture results through excessive sample decontamination or overgrowth of conventional bacteria. Oropharyngeal swabs should not be used for the detection of NTM, since they do not consistently provide sufficient material for culture.^77^

A staged approach should be adopted for obtaining diagnostic samples; testing spontaneously expectorated or induced sputum (if available) before resorting to bronchoscopy. Although there are no published studies comparing the relative performance of these different methods for detection of NTM, the presence of negative sputum samples in individuals with radiological and clinical suspicion of NTM disease should prompt CT-guided bronchoscopic sampling, as, for example, in nodular bronchiectatic disease.^78–80^ While trans-bronchial biopsies can reveal NTM (on microscopy or culture) and may demonstrate granulomatous inflammation (supporting NTM disease rather than transient colonisation), they should not be obtained routinely in individuals with CF given the significant risks of bleeding and pneumothorax.^81^

### How should respiratory tract samples from individuals with CF be cultured for NTM?

***Recommendation 9*: The CF Foundation and the ECFS recommend that respiratory tract samples should be cultured using both solid and liquid media.**

***Recommendation 10*: The CF Foundation and the ECFS recommend that the incubation duration for NTM cultures should be for a minimum of 6 weeks.**

***Recommendation 11*: The CF Foundation and the ECFS recommend that an NTM culture should be processed within 24 h of collection to optimise the detection of NTM in respiratory samples. If a delay in processing is anticipated, refrigeration of samples is advised.**

The most sensitive and rapid way to detect viable mycobacteria is to culture samples (following decontamination to remove conventional bacteria and fungi) in liquid media using an automated growth detection system (such as Mycobacteria Growth Indicator Tube (MGIT)^77^ ^82^ ^83^); a process widely used around the world. However, concomitant culture on solid media may increase the diagnostic yield since NTM can be detected despite incomplete sample decontamination.^84^ Since decontamination procedures substantially reduce the viability of mycobacteria in samples, attempts have been made to use highly selective agar for solid culture of unprocessed sputum. A recent study, using agar designed for *Burkholderia cepacia* complex culture,^84^ demonstrated an improvement in detection of rapidly growing mycobacteria from 0.7% with conventional liquid culture to 2.8%. The duration, both of liquid and solid culture methods, has not been rigorously tested but the vast majority of pathogenic NTM will grow by 6 weeks—the current recommended duration in US and European laboratories.^77^

Laboratory processing of samples should ideally be performed within 24 h of collection to avoid overgrowth by conventional bacteria, which can reduce NTM viability^85^ and prevent successful decontamination.^85^ Studies have shown that refrigeration of samples may improve NTM detection from sputum samples^86^ and should be considered if delays longer than 24 h in processing are anticipated.

### How should respiratory tract samples from individuals with CF be decontaminated to optimise the detection of NTM?

***Recommendation 12*: The CF Foundation and the ECFS recommend that respiratory tract samples should be decontaminated using the standard *N*-acetyl l-cysteine, NALC, (0.5%)-NaOH (2%) method.**

***Recommendation 13*: The CF Foundation and the ECFS recommend that, if a sample remains contaminated with Gram-negative bacteria after standard NALC-NaOH decontamination, it should be further treated with either 5% oxalic acid or 1% chlorhexidine.**

Adequate sample decontamination to remove conventional bacteria and fungi is essential to permit culture-based detection of mycobacteria,^77^ ^87^ ^88^ but often fails in CF samples given high densities of *P. aeruginosa* and other microbes.^39–41^ ^89^ ^90^ Since enhanced decontamination protocols adversely impact on NTM viability in samples,^90^ a two-step approach to sample processing should be adopted.^41^ Virtually all US and European clinical microbiology laboratories currently use an NALC-NaOH decontamination step prior to mycobacterial culture.^41^ ^87^ ^88^

The addition of a second decontamination step using oxalic acid has been shown to permit the recovery of NTM from persistently contaminated samples albeit with reduced sensitivity.^40^ Alternatively, use of 1% chlorhexidine as a first step may improve the recovery of mycobacteria, but at the expense of higher rates of residual sample contamination.^89^ Chlorhexidine negatively affects the performance of the MGIT automated liquid culture system, because it needs to be neutralised with lecithin; lecithin generates random fluorescence reactions from the MGIT system sensor, limiting its use.^89^

### Should non-culture-based methods be used to detect NTM in respiratory tract samples from individuals with CF?

***Recommendation 14*: The CF Foundation and the ECFS recommend against the use of non-culture-based methods for detecting NTM in respiratory tract samples.**

A number of studies have been published on the use of PCR-based detection methods for NTM from respiratory samples.^91–95^ To date, however, none have been robustly evaluated for CF sputum samples, nor have they demonstrated sufficiently high sensitivity and specificity on smear-negative samples^91^ to recommend their routine diagnostic use. Furthermore, the clinical significance of PCR-positive respiratory samples is currently unknown.

### How should NTM isolates from individuals with CF be identified?

***Recommendation 15*: The CF Foundation and the ECFS recommend that all NTM isolates from individuals with CF should undergo molecular identification.**

***Recommendation 16*: The CF Foundation and the ECFS recommend that all NTM isolates from individuals with CF should be identified to the species level, except for *M. intracellulare*, *M. avium and M. chimaera*, where identification can be limited to MAC and MABSC, which should be subspeciated.**

As individual NTM species differ in their potential to cause clinical disease in humans^96^ and in their response to specific antibiotics, correct species identification of NTM isolates is clinically important. Moreover, in the case of *M. abscessus*, the ability to identify isolates to the subspecies level (*M. a. abscessus, M. a. bolletii*, *M. a. massiliense*) may predict treatment response^97^ and potentially permit targeted therapy.^98^ *M. a. massiliense* harbours a partial *erm*41 gene deletion, preventing inducible macrolide resistance,^97^ ^99^ and leads to more successful outcomes with macrolide-based antibiotic regimens than in infections with *M. a. abscessus* (which has a full length, functional *erm*41 gene).^97^

There is no gold standard for NTM species identification. Molecular methods have now surpassed biochemical tests for NTM identification in many laboratories.^100–107^ Although matrix-assisted laser desorption ionisation-time of flight mass spectrometry has shown promise in providing rapid speciation of NTM,^108–112^ the optimal method for protein extraction from mycobacteria and the exact discriminatory power of this method have yet to be established.

Among molecular methods, three techniques are in current clinical use. The first includes line probe assays,^103–105^ ^113^ which are easy to perform but costly, and permits accurate identification of the most frequently encountered NTM species but not subspeciation of *M. abscessus*. The second technique is PCR product restriction analysis in which amplified gene fragments are restriction digested to yield different sized fragments, which are then resolved by gel electrophoresis and correlated with specific species.^114^ This technique is mostly used in low-resource settings and is at least comparable to the line probe assays.^106^ The third technique is (partial) gene sequencing, which permits a higher level of discrimination, often to subspecies level, but is only available in laboratories with access to sequencing facilities. The choice of the optimal sequencing strategy is not straightforward. Although partial 16S ribosomal RNA (rRNA) gene sequencing provides insufficient discrimination, particularly between *M. abscessus* and *M. chelonae*,^115^ a number of other gene sequences (such as partial *hsp65* and *rpoB* gene sequences) have been successfully used.^107^ ^116^ For subspeciation of *M. abscessus*, a multilocus sequence typing approach has recently been validated.^116–118^ An alternative strategy close to subspeciation is to measure *erm* gene associated inducible macrolide resistance by phenotypic drug susceptibility testing (DST). This does not distinguish accurately between *M. abscessus* subspecies but does offer the data for which the subspeciation is generally performed—whether or not there is inducible macrolide resistance.

### Should DST be performed on NTM isolates from individuals with CF?

***Recommendation 17*: The CF Foundation and the ECFS recommend that for MAC, clarithromycin susceptibility testing should be performed on an isolate recovered prior to initiation of treatment. Clarithromycin susceptibility testing should also be performed on subsequent isolates if the patient (a) fails to culture convert after 6 months of NTM treatment; (b) recultures MAC after initial culture conversion while on NTM treatment or (c) recultures MAC after completion of NTM treatment.**

***Recommendation 18*: The CF Foundation and the ECFS recommend that for MABSC, susceptibility testing should include at least clarithromycin, cefoxitin and amikacin (and preferably also tigecycline, imipenem, minocycline, moxifloxacin and linezolid).**

***Recommendation 19*: The CF Foundation and the ECFS recommend that DST should be performed in accordance with CLSI guidelines.**

Based on current published data, the exact role of DST and its potential to guide regimen selection and predict outcomes in NTM lung disease in patients with CF, remains unknown.^119^ The CLSI has published guidelines on DST of NTM.^17^ ^120^ ^121^ Its European counterpart, the European Committee on Antimicrobial Susceptibility Testing (EUCAST), presently has no guidelines for DST of NTM.^77^

It is important to appreciate that, although CLSI guidelines provide breakpoint concentrations to interpret minimum inhibitory concentrations (MICs) as ‘susceptible’ or ‘resistant’, these cut-offs have had very limited clinical validation, and no clinical validation has been performed in patients with CF. Moreover, limited pharmacokinetic (PK) data are now available for MAC lung disease to support breakpoint concentrations,^122^ there are no representative PK or pharmacodynamic data to guide treatment of patients with CF.

Breakpoints for clarithromycin susceptibility of MAC have been validated in HIV-related disseminated MAC disease and in retrospective series of MAC lung disease.^119^ ^123^ ^124^ Since the presence of macrolide resistance predicts worse clinical outcomes^125^ ^126^ and requires augmented treatment,^126^ susceptibility to macrolides should be tested on isolates prior to treatment initiation and during treatment in refractory cases defined as those individuals who (1) fail to culture convert after 6 months of NTM treatment; (2) reculture MAC after initial culture conversion while on NTM treatment or (3) reculture MAC after completion of NTM treatment**.**

A very recent study has shown that amikacin MICs >64 mg/L are measured only in MAC isolates that have mutations associated with amikacin resistance, that is, in the 16S rRNA gene. These strains are cultured from patients with significant aminoglycoside exposure, such as individuals with CF, and for disease caused by these strains, amikacin is unlikely to have any beneficial effect.^127^

For rapidly growing mycobacteria including *M. abscessus*, clinical validation has only been performed in series of extra-pulmonary disease,^128^ and only for cefoxitin, aminoglycosides and co-trimoxazole. In series of *M. abscessus* lung disease, the outcomes of macrolide-based treatment are generally poor and do not correlate well with in vitro susceptibilities^119^ ^129^ potentially due to *erm*^41^-dependent inducible macrolide resistance and relative short duration of adequate regimens, which were often interrupted because of toxicity. Indeed, in the absence of a functional *erm*^41^ gene, response to macrolide-containing treatments has been good.^94^ The CLSI has recommended routine testing for inducible macrolide resistance by performing extended incubation of isolates in the presence of clarithromycin, as inducible resistance may predict treatment failure.^120^ For *M. simiae*, the role of DST is unknown, although the generally poor outcomes of treatment have been correlated with a lack of synergistic activity between rifampicin and ethambutol, an in vitro observation that still awaits clinical validation.^130^ Some molecular methods to assess drug susceptibility exist, but are not yet routinely available. For example, sequencing of the 16S rRNA and 23S rRNA genes can reveal mutations associated with high-level resistance to aminoglycosides and macrolides, respectively.^119^ ^127^

## Diagnosis of NTM-PD in CF

### Should the ATS/IDSA criteria for the diagnosis of NTM-PD be used in individuals with CF?

***Recommendation 20*: The CF Foundation and the ECFS recommend that ATS/IDSA criteria for the diagnosis of NTM-PD should be used in individuals with CF (ATS/IDSA 2007 Statement).**

***Recommendation 21*: The CF Foundation and the ECFS recommend that other CF pathogens and comorbidities should be considered as potential contributors to a patient's symptoms and radiological features when determining the clinical significance of NTM-positive cultures.**

***Recommendation 22*: The CF Foundation and the ECFS recommend that NTM treatment should be considered for individuals with CF who have ATS/IDSA defined NTM-PD.**

***Recommendation 23*: The CF Foundation and the ECFS recommend that individuals receiving azithromycin as part of their CF medical regimen who have a positive NTM culture should not continue azithromycin treatment while evaluation for NTM disease is underway, as azithromycin monotherapy may lead to resistance. A macrolide agent may be included in a multidrug treatment regimen if criteria are met for NTM disease.**

In contrast to *M. tuberculosis*, a single positive culture of NTM does not necessarily indicate that an individual has NTM-PD. To address the difficulty of making a diagnosis of NTM-PD, the ATS/IDSA proposed a set of clinical, radiological and microbiological criteria required to define an individual as having NTM-PD (ref 22; box 1). Although these criteria have not been validated for individuals with CF, they have been widely adopted by NTM specialists around the world and provide an operational definition for NTM-PD, which supports clinical decision-making and facilitates research. The Statements Committee therefore concluded that, in the absence of an alternate, CF-validated definition, the ATS/IDSA criteria should be used for the definition of NTM-PD in individuals with CF.

Box 1 **ATS/IDSA clinical and microbiologic criteria for diagnosing non-tuberculous mycobacterial pulmonary disease (NTM-PD) (based on ref 22)**

Clinical (both required)

1. Pulmonary symptoms with nodular or cavitary opacities on chest radiograph, or a high-resolution CT scan that shows multifocal bronchiectasis with multiple small nodules.
2. Appropriate exclusion of other diagnoses.

Microbiologic (one of the following required)

- Positive culture results from at least two expectorated sputum samples. If the results from samples are non-diagnostic, consider repeat sputum acid-fast bacilli (AFB) smears and cultures.
- Positive culture results from at least one bronchial wash or lavage.
- Transbronchial or other lung biopsy with mycobacterial histopathological features (granulomatous inflammation or AFB) and positive culture for NTM or biopsy showing mycobacterial histopathological features (granulomatous inflammation or AFB) and one or more sputum or bronchial washings that are culture positive for NTM.
- Expert consultation should be obtained when either infrequently encountered NTM or those usually representing environmental contamination are recovered.
- Patients who are suspected of having NTM-PD but who do not meet the diagnostic criteria should be followed until the diagnosis is firmly established or excluded.
- Making the diagnosis of NTM-PD does not, per se, necessitate the institution of therapy, which is a decision based on potential risks and benefits of therapy for individual patients.

#### Microbiological criteria for NTM-PD

Individuals should have two or more positive sputum cultures of the same NTM species or one positive culture from bronchoscopic lavage or wash. The threshold for the number of positive sputum samples is derived from an observational study of individuals without CF with MAC in which 98% individuals with at least two positive sputum cultures developed progressive radiographic change compared to only 2% with one positive culture.^131^ The type of NTM species isolated is also important. Thus, isolation of *M. abscessus* is more likely to reflect NTM-PD than culturing usually non-pathogenic species such as *M. gordonae* and *M. terrae* complex.

#### Radiological criteria for NTM-PD

In the context of CF-related lung disease, a chest radiograph is unlikely to be of use for the investigation of NTM-PD. High-resolution CT (HRCT) scan changes supporting a diagnosis of NTM-PD would include: inflammatory nodules, new tree-in-bud opacities (particularly in areas of mild underlying bronchiectasis) and cavitation.^132^ However, these changes are non-specific, particularly in individuals with severe CF-related lung disease, and may reflect infection with more common CF pathogens, inadequate airway clearance or the development of allergic bronchopulmonary aspergillosis (ABPA).

#### Clinical criteria for NTM-PD

NTM-PD should be suspected in individuals with worsening respiratory symptoms (breathlessness, increased cough and sputum production) and/or declining pulmonary function tests that do not respond to antibiotic therapy targeting conventional CF-associated bacteria and optimised airway clearance. Night sweats, fevers, chest pains and weight loss (although uncommon) may also suggest possible NTM-PD.

NTM treatment should be considered in individuals with CF who fulfil ATS/IDSA criteria for NTM-PD. However, the decision to start treatment is a clinical one based on an amalgamation of patient factors, the NTM species involved, the risks of treatment side effects, adherence concerns and the expected outcomes of treatment.

### Recommended clinical practice for diagnosis

**Figure 1:**
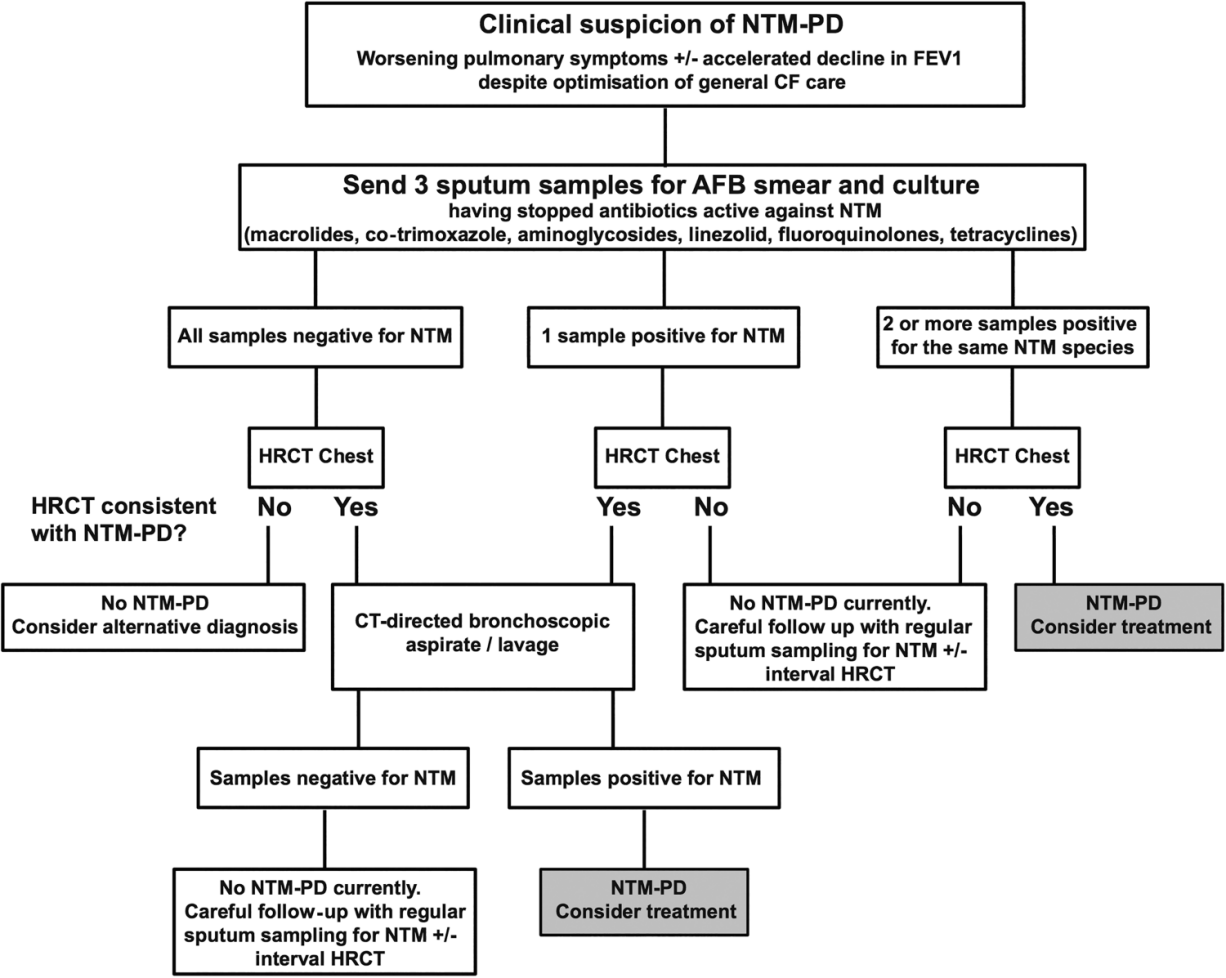
A suggested algorithm for the investigation of individuals with clinical suspicion of NTM-PD (AFB, acid-fast bacilli; CF, cystic fibrosis; FEV_1_, forced expiratory volume in 1 s; HRCT, high-resolution CT; NTM-PD, non-tuberculous mycobacteria pulmonary disease).

A suggested algorithm for the investigation of individuals with CF suspected of having NTM-PD is shown in figure 1.

When being investigated for potential NTM-PD, individuals should discontinue drugs liable to compromise NTM culture (such as macrolides, fluoroquinolones, aminoglycosides, co-trimoxazole, linezolid and doxycycline) prior to sputum sample collection. In the case of azithromycin, intracellular accumulation within phagocytes may require a washout period of 2 weeks or more to allow for drug clearance.^133^ ^134^ If sputum samples are persistently culture negative, but clinical or radiological suspicion of NTM-PD remains, bronchoscopy with targeted sampling of areas with suggestive HRCT changes may be indicated. Individuals receiving azithromycin as part of their CF medical regimen, who have a positive surveillance NTM culture, should not continue azithromycin treatment while evaluation for NTM disease is underway, as azithromycin monotherapy may lead to the development of macrolide resistance.

Other CF pathogens and comorbidities should be considered as potential contributors to a patient's symptoms and radiological features when determining the clinical significance of NTM-positive cultures. All aspects of CF care should be reviewed and optimised in order to determine the clinical significance of NTM in the sputum. Specifically, consider a trial of NTM-sparing intravenous antibiotics (ie, avoid carbapenems, cefoxitin, tigecycline, fluoroquinolones, linezolid and amikacin) that target conventional bacteria; and assess for CF-related diabetes, uncontrolled gastrointestinal reflux disease, and clinical and immunological features of ABPA. Likewise, adequate treatment of sinus disease, nutritional support and effective airway clearance strategies should be implemented.

Before starting NTM treatment, side effects, the importance of adherence to therapy and complications of treatment should be discussed with patients, and these discussions documented in the medical notes. Discussion of the risk of treatment failure should be clearly documented.

## Treatment

### Which antibiotic regimen should be used in individuals with CF who have ATS/IDSA-defined MABSC pulmonary disease?

***Recommendation 24*: The CF Foundation and the ECFS recommend that treatment of MABSC pulmonary disease should involve an intensive phase followed by a continuation phase.**

***Recommendation 25*: The CF Foundation and the ECFS recommend that the intensive phase should include a daily oral macrolide (preferably azithromycin) in conjunction with 3–12 weeks of intravenous amikacin and one or more of the following: intravenous tigecycline, imipenem or cefoxitin, guided but not dictated by DST. The duration of intensive phase therapy should be determined by the severity of infection, the response to treatment and the tolerability of the regimen.**

***Recommendation 26*: The CF Foundation and the ECFS recommend that the continuation phase should include a daily oral macrolide (preferably azithromycin) and inhaled amikacin, in conjunction with 2–3 of the following additional oral antibiotics: minocycline, clofazimine, moxifloxacin and linezolid, guided but not dictated by DST.**

***Recommendation 27*: The CF Foundation and the ECFS recommend that individuals with MABSC pulmonary disease should be managed in collaboration with experts in the treatment of NTM and CF, as drug intolerance and drug-related toxicity occur frequently, and changes in antibiotic therapy are often required.**

***Recommendation 28*: The CF Foundation and the ECFS recommend that monotherapy with a macrolide or other antimicrobial should never be used in the treatment of MABSC pulmonary disease.**

There are no published randomised controlled trials evaluating treatment outcomes in individuals with *M. abscessus* pulmonary infections. Current treatment recommendations from the ATS and IDSA recommend consideration of a multidrug treatment regimen, but note that long-term sputum conversion is difficult to achieve and thus, alternative goals such as symptomatic improvement, radiographic regression of opacities or microbiological improvement, may be more realistic.^26^ The ATS/IDSA recommendations were based primarily on a single large study of 154 patients with lung disease caused by rapidly growing mycobacteria, in which more than 80% of patients were infected by *M. abscessus.*^135^ Treatment outcomes were extremely poor; however, the patients did not receive the currently recommended combination of antibiotics.

Since the publication of the last ATS/IDSA guidelines,^26^ there have been several studies that reported treatment outcomes in individuals without CF with pulmonary disease due to *M. abscessus*. Jeon *et al*^136^ described treatment outcomes in 65 non-CF adults, in South Korea, with *M. abscessus* lung disease, who received a standardised treatment regimen. The regimen included 4 weeks of amikacin (15 mg/kg/day in two divided doses) and cefoxitin (200 mg/kg/day in three divided doses) along with clarithromycin (1000 mg/day in two divided doses), ciprofloxacin (1000 mg/day in two divided doses) and doxycycline (200 mg/day in two divided doses). The total duration of therapy was 24 months and at least 12 months after sputum culture conversion. Fifty-four (83%) patients responded with improved symptoms and 48 (74%) with improved HRCT findings. Sputum conversion and maintenance of negative sputum cultures for more than 12 months was achieved in 38 (58%) patients. This rate was significantly lower (17%) in patients whose isolates were resistant to clarithromycin. In contrast, in the 14 (22%) patients who underwent resectional surgery, negative sputum cultures were achieved and maintained in 7 (88%) of 8 with preoperatively positive cultures. The authors concluded that a standardised regimen was moderately effective, but adverse reactions were frequent.

Among 107 patients with *M. abscessus* pulmonary infection at National Jewish Health in Denver, CO, 69 non-CF individuals were treated and followed for a mean duration of 34 months.^129^ Patients were treated with individualised treatment regimens following ATS/IDSA recommendations. Twenty (29%) patients remained culture positive, 16 (23%) converted but experienced relapse, 33 (48%) converted to negative and did not relapse, while 17 (16%) died during the study period. There were significantly more surgical patients than medical patients whose culture converted and remained negative for at least 1 year (57% vs 28%, p=0.022). As in the previous study from South Korea, surgery may have been beneficial. However, surgical management is less likely to be applicable in individuals with CF in whom focal pulmonary disease is uncommon.

In a follow-up study, Koh *et al*^97^ reported significant differences in outcomes based on which subspecies of *M. abscessus* was causing the infection. Treatment response rates to a standardised multidrug regimen were much higher in patients with *M. a. massiliense* than in those with *M. a. abscessus*: sputum culture conversion occurred in 88% of patients with *M. a. massiliense* compared with 25% with *M. a. abscessus* (p<0.001). All of the *M. a. abscessus* isolates contained a full length, functional *erm*^41^ that was shown to result in inducible macrolide resistance when the isolates were incubated with clarithromycin. In contrast, the MIC of *M. a. massiliense* strains did not increase after incubation with the macrolide agent because the *erm*^41^ gene contained a deletion, making it non-functional. Recent data from this same group of investigators have indicated that clarithromycin is a much stronger inducer of *erm*^41^ than azithromycin, suggesting that the latter macrolide may be a better choice when treating *M. a. abscessus* infections.^98^

Despite the clinical significance of *M. abscessus* lung infection in patients with CF, data on treatment outcomes are extremely limited. There is one anecdotal report that describes eradication of *M. abscessus* in an individual with CF who received a prolonged course of therapy with alternating month inhaled amikacin plus oral clarithromycin.^137^ However, this appears to be an uncommon outcome in practice. A recent case series of 52 individuals, including 15 with CF, with *M. abscessus* and/or *M. chelonae* infection, suggests that tigecycline-based regimens may be of benefit, with 10/15 individuals with CF showing some improvement.^138^

### Recommended clinical practice for antibiotic treatment for *M. abscessus* pulmonary disease in CF

**Figure 2:**
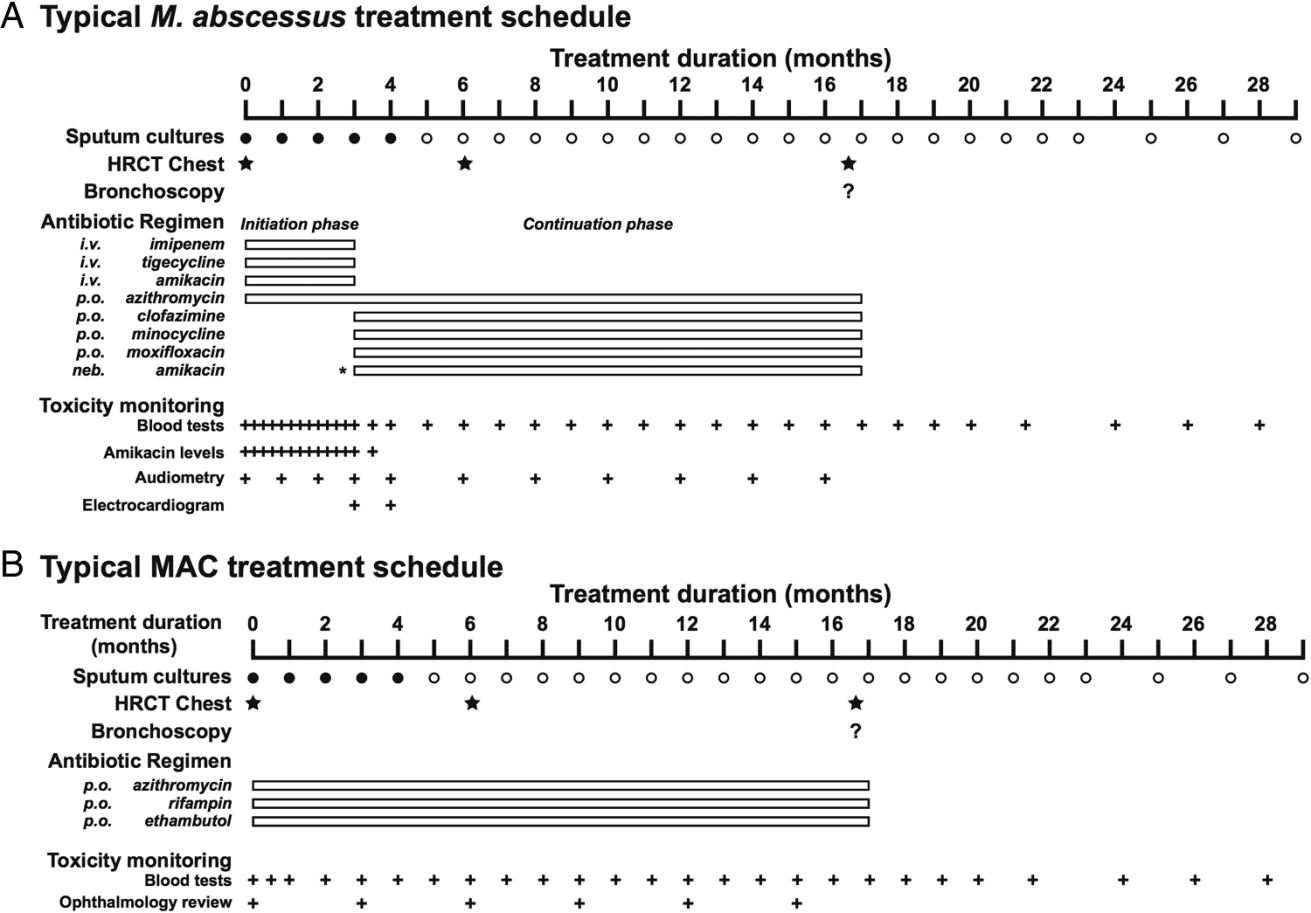
Typical treatment schedules for individuals with CF with *Mycobacterium abscessus* or MAC pulmonary disease. (A) *M. abscessus* treatment is divided into an initial intensive phase with an oral macrolide (preferably azithromycin) and intravenous amikacin with one or more additional intravenous antibiotics (tigecycline, imipenem, cefoxitin) for 3–12 weeks (depending on severity of infection, response to treatment, and the tolerability of the regimen), followed by a continuation phase of oral macrolide (preferably azithromycin) and inhaled amikacin with 2–3 additional antibiotics (minocycline, clofazimine, moxifloxacin, linezolid). Antibiotic choices should be guided but not dictated by drug susceptibility testing. Baseline and interval testing for drug toxicity is essential (B). MAC treatment (for clarithromycin-sensitive disease) should be with a daily oral macrolide (preferably azithromycin), rifampin and ethambutol. An initial course of injectable amikacin or streptomycin should be considered in the presence of (i) AFB smear positive respiratory tract samples, (ii) radiological evidence of lung cavitation or severe infection and (iii) systemic signs of illness. Baseline and interval testing for drug toxicity is essential (AFB, acid-fast bacilli; CF, cystic fibrosis; HRCT, high-resolution CT; MAC, *Mycobacterium avium* complex).

A typical treatment schedule for individuals with CF with *M. abscessus* infection is shown in figure 2. Antibiotic dosing regimens are listed in table 2 with important side effects/toxicities described in table 3.

**Table 2:** Antibiotic-dosing regimens used to treat *Mycobacterium avium* complex and *Mycobacterium abscessus* complex pulmonary disease in cystic fibrosis

Antibiotic	Route	Dose suitable for children/adolescents	Dose suitable for adults
Amikacin*	Intravenous	Children: 15–30 mg/kg/dose once daily Adolescents: 10–15 mg/kg/dose once daily Maximum dose 1500 mg daily	10–30 mg/kg once daily or 15 mg/kg/day in two divided doses Daily to 3× weekly dosing
Amikacin*†‡	Nebulised	250–500 mg/dose once or twice daily	250–500 mg once or twice daily
Azithromycin	Oral	Children: 10–12 mg/kg/dose once daily Adolescents: adult dosing regimen Maximum dose 500 mg	250–500 mg once daily
Cefoxitin	Intravenous	50 mg/kg/dose thrice daily (maximum dose 12 g/day)	200 mg/kg/day in three divided doses (maximum dose 12 g/day)
Clarithromycin	Oral	7.5 mg/kg/dose twice daily (maximum dose 500 mg)	500 mg twice daily§
Clarithromycin	Intravenous	Not recommended	500 mg twice daily§
Clofazimine†¶	Oral	1–2 mg/kg/dose once daily (maximum dose 100 mg)	50–100 mg once a day
Co-trimoxazole (sulfamethoxazole and trimethoprim)	Oral	10–20 mg/kg/dose twice daily	960 mg twice daily
Co-trimoxazole (sulfamethoxazole and Trimethoprim)	Intravenous	10–20 mg/kg/dose twice daily	1.44 g twice daily
Ethambutol	Oral	Infants and children: 15 mg/kg/dose once daily Adolescents: 15 mg/kg/dose once daily	15 mg/kg once daily
Imipenem	Intravenous	15–20 mg/kg/dose twice daily (maximum dose 1000 mg)	1 g twice daily
Linezolid**	Oral	<12 years old: 10 mg/kg/dose thrice daily 12 years and older: 10 mg/kg/dose once or twice daily (maximum dose 600 mg)	600 mg once or twice daily
Linezolid**	Intravenous	<12 years old: 10 mg/kg/dose thrice daily 12 years and older: 10 mg/kg/dose once or twice daily (maximum dose 600 mg)	600 mg once or twice daily
Moxifloxacin	Oral	7.5–10 mg/kg/dose once daily (maximum dose 400 mg daily)	400 mg once daily
Minocycline	Oral	2 mg/kg/dose once daily (maximum dose 200 mg)	100 mg twice daily
Rifampin (Rifampicin)	Oral	10–20 mg/kg/dose once daily (maximum dose 600 mg)	<50 kg 450 mg once daily >50 kg 600 mg once daily
Rifabutin	Oral	5–10 mg/kg/dose once daily (maximum dose 300 mg)	150–300 mg once daily 150 mg if patient taking strong CYP3A4 inhibitor 450–600 mg if patient taking strong CYP3A4 inducer
Streptomycin*	Intramuscular/intravenous	20–40 mg/kg/dose once daily (maximum dose 1000 mg)	15 mg/kg once daily (maximum dose 1000 mg)
Tigecycline†,††	Intravenous	8–11 years: 1.2 mg/kg/dose twice daily (maximum dose 50 mg) 12 years and older: 100 mg loading dose and then 50 mg once or twice daily	100 mg loading dose and then 50 mg once or twice daily

*Adjust dose according to levels. Usually, starting dose is 15 mg/kg aiming for a peak level of 20–30 µg/mL and trough levels of <5–10 micrograms/ml.

†As tolerated.

‡Mixed with normal saline.

§For individuals under 55 kg, many practitioners recommend 7.5 mg/kg twice daily.

¶Only available in the USA through an IND application to the FDA.

**Usually given with high dose (100 mg daily) pyridoxine (vitamin B_6_) to reduce risk of cytopaenias.

††Many practitioners recommend pre-dosing with one or more anti-emetics before dosing and/or gradual dose escalation from 25 mg daily to minimise nausea and vomiting.

IND, investigational new drug; FDA, Food and Drug Administration.

**Table 3:** Important side effects/toxicities of antibiotics and advisable monitoring procedures for MAC and MABSC in CF

Drug	Common side effects/toxicity	Monitoring procedures
Amikacin	Nephrotoxicity	Regular serum amikacin levels* Regular serum creatinine levels
	Auditory-vestibular toxicity (tinnitus, high-frequency hearing loss)	Symptoms, baseline and interval audiograms
Azithromycin	Nausea, vomiting, diarrhoea	Symptoms
	Auditory-vestibular toxicity	Symptoms, audiogram
	Prolonged QT	ECG
Clarithromycin	Hepatitis	Liver function tests
	Taste disturbance	Symptoms
	Inhibited hepatic metabolism of rifabutin	Symptoms
Cefoxitin	Fever, rash	Symptoms
	Eosinophilia, anaemia, leucopaenia, thrombocytopaenia	Full blood count
	Interference with common assays to measure serum creatinine	Use alternative assay
Clofazimine	Discoloration of skin†	Symptoms
	Enteropathy (sometimes mimicking pancreatic insufficiency)†	Symptoms
	Nausea and vomiting	Symptoms
Co-trimoxazole	Nausea, vomiting, diarrhoea	Symptoms
	Anaemia, leucopoenia, thrombocytopaenia	Full blood count
	Fever, rash, Stevens-Johnson syndrome	Symptoms
Ethambutol	Optic neuritis	Symptoms (loss of colour vision/acuity) Baseline and interval testing for colour vision and acuity‡ Ophthalmology opinion if symptoms occur
	Peripheral neuropathy	Symptoms; nerve conduction studies
Imipenem	Hepatitis	Liver function tests
Imipenem (cont)	Nausea, vomiting, diarrhoea	Symptoms
Linezolid	Anaemia, leucopaenia, thrombocytopaenia	Full blood count
	Peripheral neuropathy	Symptoms/clinical evaluation/electrophysiology
	Optic neuritis	Symptoms (loss of colour vision/acuity) Baseline and interval testing for colour vision and acuity Ophthalmology opinion if symptoms occur
Moxifloxacin	Nausea, vomiting, diarrhoea	Symptoms
	Insomnia, agitation, anxiety	Symptoms
	Tendonitis	Symptoms
	Photosensitivity	Symptoms
	Prolonged QT	ECG
Minocycline	Photosensitivity	Symptoms
	Nausea, vomiting, diarrhoea	Symptoms
	Vertigo	Symptoms
	Skin discolouration	Clinical evaluation
Rifampin and rifabutin	Orange discolouration of bodily fluids (can stain contact lenses)	Symptoms
	Hepatitis	Liver function tests
	Nausea, vomiting, diarrhoea	Symptoms
	Fever, chills	Symptoms
	Thrombocytopaenia	Full blood count
	Renal failure (rifampin)	Blood tests
	Increased hepatic metabolism of numerous drugs	Dose adjustment of other medications/serum levels where available
Rifabutin	Leucopaenia,	Full blood count
	Anterior uveitis (when combined with clarithromycin)	Symptoms
	Flu-like symptoms polyarthralgia, polymyalgia	Symptoms
Streptomycin	Nephrotoxicity	Regular serum streptomycin levels Regular serum creatinine levels
	Auditory-vestibular toxicity (tinnitus, high frequency hearing loss)	Symptoms, baseline and interval audiograms
Tigecycline	Nausea, vomiting, diarrhoea	Symptoms
	Pancreatitis	Serum amylase§
	Hypoproteinaemia	Serum albumin
	Bilirubinaemia	Serum bilirubin

*Usually aiming for peak levels of 20–30 µg/mL and trough levels of <5–10 µg/mL.

†It may take up to 3 months for toxicity to resolve following cessation of clofazimine due to its long half-life.

‡Monthly checks if receiving 25 mg/kg/day.

§In individuals with pancreatic sufficiency.

CF, cystic fibrosis; MABSC, *Mycobacterium abscessus* complex; MAC, *Mycobacterium avium* complex.

Given the lack of clinical trial data to inform treatment decisions there is a lot of variation in how patients are treated. An initial intensive phase is typically used to rapidly decrease the bacterial load. A combination of two intravenous drugs with demonstrated in vitro activity is administered for several weeks to months in combination with one or more oral drugs. Intravenous drug regimens of amikacin with cefoxitin and/or imipenem and/or tigecycline are the most commonly used combinations. Oral drugs with demonstrated in vitro activity include the macrolides (clarithromycin and azithromycin), linezolid, clofazimine and, occasionally, ciprofloxacin and/or moxifloxacin. After the intensive phase of therapy, patients are usually treated with at least two oral drugs in addition to a macrolide with or without inhaled antibiotics.

However, there is growing concern that treatment of *M. abscessus* isolates that have either a functional *erm*^41^ gene (resulting phenotypically in inducible macrolide resistance) or a 23S rRNA mutation (leading to high level constitutive macrolide resistance) may be compromised by switching from intravenous to oral therapy (given the relatively poor efficacy of oral antibiotics) and, therefore, continuous/very extended intravenous therapy with two or more effective antibiotics may be indicated in these cases.

The choice of intravenous agents is based on in vitro activity and the toxicity profile of the drug. In addition to amikacin, imipenem is perhaps the best choice as companion intravenous therapy; the drug shows in vitro activity and the side effect profile is better than that of cefoxitin and tigecycline. In the study reported by Jeon *et al*,^136^ 60% of the patients started on cefoxitin had to have the drug discontinued due to drug-related toxicity, after a median of 22 days of treatment. Neutropaenia occurred in 51% and thrombocytopaenia in 6% of patients on cefoxitin. Tigecycline has a low MIC against *M. abscessus* and showed efficacy against *M. abscessus* in combination.^138^ However, it is associated with significant nausea and vomiting, which has made it difficult to administer for a prolonged period.^138^

There are few oral drugs with significant in vitro activity against *M. abscessus*; the macrolides are the only oral drugs with consistent activity although their use may be potentially limited by inducible resistance (as described above) or acquired point mutations in the 23S rRNA. There are no clinical trials comparing azithromycin to clarithromycin in *M. abscessus* infection, so the choice of which macrolide to use is typically based on the in vitro activity, side effects profile and consideration of drug interactions. Clarithromycin has slightly better in vitro activity than azithromycin but there are conflicting reports regarding the impact of erm^41^ gene expression with each of these drugs.^98^ ^139^ ^140^ Clarithromycin is a stronger inhibitor of the P450 enzyme system than is azithromycin, so drug interactions are more common.

Linezolid shows in vitro activity in approximately 50% of *M. abscessus* isolates (although there is considerable geographical variation); however, haematological (anaemia, thrombocytopaenia) and neurological (peripheral neuropathy, optic neuritis) toxicities are common, particularly when linezolid is dosed 600 mg two times a day for prolonged courses. For this reason, many practitioners give 600 mg once daily to reduce the risk of adverse effects. However, care should be exercised in individuals chronically co-infected with methicillin-resistant *Staphylococcus aureus* (MRSA) since long-term linezolid therapy may encourage MRSA resistance. The fluoroquinolones and minocycline/doxycycline rarely show in vitro activity although they were included in the standardised treatment regimen used in the report by Jeon *et al*.^98^ Finally, clofazimine has significant in vitro activity against *M. abscessus.*^141^ However, this drug, used to treat leprosy, is not readily available in the USA at this time, although it can be obtained with an IRB-approved protocol through submission of an individual patient use IND to the Food and Drug Administration. Instructions for this process can be found on the NTM Info and Research, Inc, website (http://www.ntminfo.org/clofazimine).

The lack of oral antibiotics with activity against *M. abscessus* has led clinicians to use inhaled amikacin, usually during the continuation phase of therapy. There are no studies correlating treatment outcomes in patients with *M. abscessus* infection with the dose of inhaled amikacin and, therefore, there is a great deal of variation in the dose used (250–500 mg), and frequency of administration (daily to twice daily). A recent study, targeting treatment refractory NTM patients, most of whom were without CF with *M. abscessus*, evaluated the effect of adding inhaled amikacin to their oral and/or intravenous drug regimens.^142^ Among the 20 patients with persistently positive cultures, 8 (40%) had at least one negative culture and 5 (25%) had persistently negative cultures after addition of inhaled amikacin. Inhaled amikacin was stopped in 7 (35%) due to toxicity. There is currently significant interest in the potential use of a liposomal formulation of amikacin (which may improve drug delivery within the lung and into infected macrophages) as part of a multidrug regimen for both *M. abscessus* and MAC. Large multicentre studies are ongoing.

The optimum duration of therapy is not known. Based on studies in individuals without CF, even prolonged treatment regimens were associated with high rates of failure and recurrence. Many patients who do not convert their cultures to negative on therapy may still benefit from continuing or repeating courses of treatment.

### Treatment for MAC

#### Which antibiotic regimen should be used in individuals with CF who have ATS/IDSA-defined MAC pulmonary disease?

***Recommendation 29*: The CF Foundation and the ECFS recommend the same antibiotic regimen for treatment of all species within the MAC.**

***Recommendation 30*: The CF Foundation and the ECFS recommend that clarithromycin-sensitive MAC pulmonary disease should be treated with a daily oral antibiotic regimen containing a macrolide (preferably azithromycin), rifampin and ethambutol.**

***Recommendation 31*: The CF Foundation and the ECFS recommend against the use of intermittent (three times per week) oral antibiotic therapy to treat MAC pulmonary disease.**

***Recommendation 32*: The CF Foundation and the ECFS recommend that monotherapy with a macrolide or other antimicrobial agent should never be used in the treatment of MAC pulmonary disease.**

***Recommendation 33*: The CF Foundation and the ECFS recommend that an initial course of intravenous amikacin should be considered for the treatment of MAC pulmonary disease in the presence of one or more of the following: (i) AFB smear positive respiratory tract samples, (ii) radiological evidence of lung cavitation or severe infection and (iii) systemic signs of illness.**

***Recommendation 34*: The CF Foundation and the ECFS recommend that clarithromycin-resistant MAC pulmonary disease should be managed in collaboration with experts in the treatment of NTM and CF.**

There are very few published randomised controlled trials evaluating treatment for MAC pulmonary disease (MAC-PD) in non-HIV-positive patients and none in individuals with CF. In the pre-macrolide era, a UK trial of individuals without CF and with largely cavitary disease reported that those randomised to receive rifampin and ethambutol had a combined failure/relapse rate of 41% compared to 16% of patients randomised to receive rifampin, ethambutol and isoniazid (p=0.033).^143^ In a subsequent study on a similar cohort, patients randomised to receive rifampin, ethambutol and clarithromycin had an all-cause mortality of 48% compared to 30% of patients randomised to receive rifampin, ethambutol and ciprofloxacin.^144^ However, only 13% of patients in the clarithromycin group failed treatment or relapsed compared to 23% in the ciprofloxacin group.

In addition, there have been several non-comparator studies evaluating outcomes in HIV-negative patients with MAC-PD. The majority utilised a three oral drug regimen including a macrolide (clarithromycin or azithromycin), a rifamycin (rifampin or rifabutin) and ethambutol, in combination with an initial course of an aminoglycoside (streptomycin, amikacin or kanamycin).^124^ ^125^ ^145–148^ The culture conversion rate varied considerably between studies (13–82%), but on the whole, in 55–65% of patients, the culture converted after 6–12 months treatment and, when reported, the mean time from starting treatment to culture conversion was 3–5 months.^124^ ^125^ Treatment failure was associated with previous MAC-PD treatment, cavitary disease, smear positivity, clarithromycin resistance at initiation of treatment, intolerance of NTM therapy and acquired clarithromycin resistance.^124^ ^125^ ^145^ ^147–149^

An alternative regimen using clofazimine with a macrolide and ethambutol in a study of 30 patients resulted in a culture conversion rate of 87% and a treatment success rate of 67%.^150^ Although 5 (19%) patients relapsed an average of 17 months after completing treatment, all MAC isolates remained clarithromycin sensitive, raising the possibility of reinfection rather than treatment failure.^151^ In another case series utilising clofazimine in combination with clarithromycin and minocycline, the culture conversion rate was 64% in patients completing the study (47% overall), which may indicate the importance of ethambutol as part of the multidrug regimen in the treatment of MAC-PD.^152^

Clarithromycin resistance developed in up to 15% of patients receiving treatment for MAC-PD and this was generally associated with clarithromycin monotherapy or the prescription of inadequate companion medications.^124–126^ ^146–149^ When taken in combination with ethambutol and a rifamycin, acquired clarithromycin resistance developed in only 12/303 (4%) of patients. In the context of clarithromycin resistance, the best treatment responses were seen in patients who underwent surgical resection and received >6 months of an injectable aminoglycoside (amikacin or streptomycin),^124–126^ as 11/14 (79%) so treated achieved culture conversion compared to 1/27 (4%) of those not surgically resected and not receiving injectables.^123^

While intermittent and daily dosing regimens appear equally effective in several case series of individuals without CF, intermittent regimens may be associated with less toxicity, better tolerability and adherence, and lower cost.^147^ ^148^ However, a large multicentre study utilising an intermittent dosing regimen in individuals with moderate or severe MAC-PD (including many with cavitary disease and with prior treatment failure), reported a culture conversion rate of only 13% after 12 months of treatment.^146^ ^151^ There have, to date, been no studies in individuals with CF to determine an optimal dosing regimen for MAC therapy, but concerns about drug absorption and lung penetration in CF have meant that many centres have adopted daily dosing protocols.

It is unclear if use of an aminoglycoside during the initial phase of MAC antibiotic therapy is beneficial. In a multicentre study involving 146 HIV-negative patients with MAC-PD, participants were randomised to receive intramuscular streptomycin (15 mg/kg) or placebo thrice weekly for the first 3 months of therapy, in addition to clarithromycin, rifampin and ethambutol. Streptomycin-treated patients had a significantly higher culture conversion rate after approximately 2 years of treatment than did placebo patients (71% vs 51%, p<0.05), but a third of patients in each group experienced sputum relapse and there were no significant differences in symptoms or radiological response between groups.^153^ Furthermore, there was no statistically significant difference in culture conversion rates between individuals who received an initial course of intramuscular kanamycin (78%) compared to those who did not (58%) in a non-randomised study involving patients with MAC-PD.^125^ Recently, the use of aerosolised amikacin in addition to standard multidrug macrolide-based regimens was reported in six HIV-negative individuals with MAC-PD who had failed standard therapy.^154^ While four patients were culture negative after 6 months of therapy, one later cultured *M. chelonae* (resistant to amikacin), two re-cultured MAC and one patient was unable to tolerate prolonged therapy with aerosolised amikacin. A recent case series of the impact of nebulised amikacin (250 mg once or twice daily)^142^ in 20 individuals without CF with treatment refractory NTM-PD (of whom 5 had MAC) reported adverse events in 35% of cases. Two patients discontinued therapy due to hearing loss. Studies examining the use of liposomal amikacin (which may have a better side effects profile) for the treatment of NTM in individuals with CF are ongoing.

#### Recommended clinical practice antibiotic treatment for MAC-PD in CF

A typical treatment schedule for individuals with CF with MAC infection is shown in figure 2. Antibiotic dosing regimens are listed in table 2 with important side effects/toxicities described in table 3.

Individuals with clarithromycin sensitive MAC-PD should be treated with a daily oral antibiotic regimen that includes a macrolide, rifampin and ethambutol (15 mg/kg), consistent with the ATS/IDSA recommendations for individuals with severe nodular bronchiectatic disease.^26^ Intermittent oral antibiotic therapy is not recommended due to the nature of the underlying lung disease and concerns regarding antibiotic absorption in CF. While there are no head to head trials showing a difference in outcome between individuals with MAC-PD treated with clarithromycin or azithromycin, the latter may be the macrolide of choice in CF, as it can be taken once daily, its serum levels may be less affected by rifamycins^122^ and it has well established benefits in individuals with CF in addition to its effects on NTM.

Individuals with a high bacterial load (suggested by smear positivity, radiological evidence of lung cavitation and/or significant inflammatory change or the presence of systemic symptoms) may benefit from an initial (1–3 month) course of injectable amikacin or streptomycin, in addition to the standard three-drug regimen for MAC-PD. While the available data do not show a difference in toxicity between amikacin regimens dosed at 15 mg/kg once daily or 25 mg/kg thrice weekly, ototoxicity was found in 37% of all participants (associated with older age and larger cumulative dose), vestibular toxicity in 8% (usually reversible) and nephrotoxicity in 15% (usually mild and reversible).^155^ Streptomycin, although less widely used for MAC-PD than amikacin, may have less ototoxicity than amikacin.^155^ The use of aerosolised amikacin in place of an intravenous aminoglycoside may be preferable in terms of reduced burden of care and toxicity, but outcome data are limited and it is unlikely to be helpful for patients with cavitary disease in whom drug levels at the site of infection may be subtherapeutic.

The major risk factors for the development of clarithromycin-resistant MAC-PD are macrolide monotherapy and prior macrolide treatment with inadequate companion medications. Thus, macrolides (often prescribed for their anti-inflammatory effects in CF) should be discontinued immediately following isolation of a mycobacterial species, and macrolides should never be prescribed in the treatment of MAC-PD without two appropriate companion antibiotics.

Macrolide therapy is not generally recommended in the context of clarithromycin-resistant MAC-PD,^26^ but macrolides may still be beneficial in this context in CF due to their non-antibiotic properties. Individuals with clarithromycin-resistant MAC-PD may respond to a regimen including a parenteral aminoglycoside, a rifamycin (usually rifabutin) and ethambutol, in addition to one or more companion medications (accepting that there are limited data to guide practice)^26^ ^126^ ^144^ such as a quinolone or clofazimine. Rifabutin may be useful in the treatment of clarithromycin-resistant MAC-PD, but adverse events (particularly blood dyscrasias, gastrointestinal upset and polyarthralgia) are more common and often necessitate dose reduction or complete cessation of treatment.^156–158^ Surgical resection might also be helpful in selected individuals with localised severe bronchiectatic disease, but this management is less likely to be useful in CF as MAC-PD is more likely to be diffuse.

Ethambutol ocular toxicity (optic or retrobulbar neuritis) may present with blurred vision, decreased acuity, central scotomas, impaired red-green colour discrimination and peripheral visual field defects. Ocular toxicity was identified in 6% of individuals without CF with MAC-PD receiving ethambutol at a dose of 25 mg/kg/day for the first 2 months followed by 15 mg/kg/day for the remainder of treatment.^159^ Ocular toxicity is more likely to occur in the context of MAC-PD than in patients receiving tuberculosis (TB) treatment due to the longer duration of therapy. While individuals prescribed ethambutol should have regular visual acuity and colour vision testing, visual symptoms often occur before measurable changes can be identified. Thus, patients should be educated about the potential side effects of ethambutol and encouraged to self-report changes in vision, following which ethambutol therapy should be discontinued until an ophthalmological assessment has taken place.

It is not uncommon for more than one NTM species to be isolated from an individual with CF.^6^ ^30^ In these circumstances, continued microbiological surveillance is advisable to determine which species is/are persistently positive and which is/are likely to be causing disease. NTM-PD is also commonly associated with ABPA and/or the identification of *Aspergillus* spp in sputum or lavage specimens. As rifamycins increase the hepatic metabolism of azole antifungal agents, the treatment of *Aspergillus* in the context of MAC-PD is more difficult. One approach is to use rifabutin in place of rifampin (as it is the rifamycin with the least cytochrome P450 enzyme induction) in conjunction with the usual companion medications for MAC and voriconazole, or posaconazole, which may be less affected by rifabutin co-medication than voriconazole is, with adjustment of drug doses according to levels.^160^ ^161^ If therapeutic drug monitoring (TDM) is not available, other approaches include using nebulised amikacin or clofazimine in place of rifampin.^150^

### Treatment: generic recommendations

#### What outcome monitoring should be performed in individuals with CF receiving treatment for NTM-PD?

***Recommendation 35*: The CF Foundation and the ECFS recommend that individuals with CF receiving NTM treatment should have expectorated or induced sputum samples sent for NTM culture every 4–8 weeks throughout the entire course of treatment to assess the microbiological response.**

***Recommendation 36*: The CF Foundation and the ECFS recommend that a schedule for detecting drug toxicity (including hearing loss, visual loss, renal impairment and liver function test abnormalities) should be set in place at the time of NTM treatment initiation and implemented throughout treatment based on the specific drugs prescribed.**

***Recommendation 37*: The CF Foundation and the ECFS recommend that an HRCT scan of the lungs should be performed shortly before starting NTM treatment and at the end of NTM treatment, to assess the radiological response.**

#### What duration of antibiotic therapy is recommended for individuals with CF receiving treatment for NTM-PD?

***Recommendation 38*: The CF Foundation and the ECFS recommend that NTM antibiotic therapy should be prescribed for 12 months beyond culture conversion (defined as three consecutive negative cultures, with the time of conversion being the date of the first of the three negative cultures) as long as no positive cultures are obtained during those 12 months.**

***Recommendation 39*: The CF Foundation and the ECFS recommend that individuals who fail to culture convert despite optimal NTM therapy may benefit from long-term suppressive antibiotic treatment.**

### Treatment: TDM

#### Should TDM be performed in individuals with CF receiving treatment for NTM-PD?

***Recommendation 40*: The CF Foundation and the ECFS recommend that, when amikacin is given intravenously or when streptomycin is given intravenously or intramuscularly, serum levels should be monitored and dosing adjusted to minimise ototoxicity and nephrotoxicity.**

***Recommendation 41*: The CF Foundation and the ECFS recommend against routinely obtaining serum levels of other anti-mycobacterial drugs. However, absorption of oral medications is often reduced in CF. Therefore use of TDM should be considered for individuals failing to improve despite taking recommended drug regimens or for those on concomitant medications with significant interactions with NTM drugs.**

TDM seeks to quantify the relationship between drug dose, serum (plasma) concentration and clinical response,^162^ and to thereby maximise therapeutic response while avoiding toxicity. The potential benefits of TDM during NTM treatment in individuals with CF include adjusting drug dosing to:

- *Correct for drug–drug interactions that could adversely affect serum antibiotic levels*: Drug–drug interactions frequently occur among agents used to treat NTM. Rifampin (more than rifabutin) may increase the metabolism of several drugs including clarithromycin, azithromycin and moxifloxacin, while rifabutin increases azithromycin levels and decreases moxifloxacin levels.^122^ ^163^
- *Maximise the PK and pharmacodynamic* *(PD) parameters of antibiotics to optimise efficacy*: The PK/PD indices that correlate with clinical efficacy vary by antimicrobial agent.^122^ ^164^ ^165^ To exert maximal activity, drugs such as aminoglycosides and ethambutol require high peak concentrations relative to the pathogen's minimal inhibitory concentration (C_max_/MIC). Ciprofloxacin and rifampin require a high concentration time or area under the plasma concentration curve measured over 24 h to MIC ratio (AUC_0–24_/MIC) and β-lactam agents require as much time as possible whereby the concentration persists above the infecting organism's MIC (%T> MIC). Macrolide agents such as azithromycin have weak concentration-dependent effects and time effects, but these agents exert their activity through intracellular activity, tissue penetration and prolonged, persistent effects, due to their long half-life.^166^
- *Overcome CF-related differences in absorption, distribution and clearance of drugs*: Individuals with CF have different renal and non-renal clearance of several drugs when compared to individuals without CF, due to reduced bioavailability, increased volume of distribution and more rapid clearance. In addition, hepatic disease and diabetes may further influence drug metabolism and absorption. Several recent reviews have addressed evidence-based dosing for various agents used for treatment of pulmonary exacerbations in CF.^167–170^ While the relevance of the recommended dosing schedules is unknown for treatment of NTM, it is possible that individuals with CF would need higher dosages of mycobacterial drugs.

With the exception of aminoglycosides, the clinical utility of TDM during treatment for NTM is unknown for individuals with and without CF due to a lack of rigorous studies, although some experts have recommended TDM for mycobacterial agents on a case-by-case basis.^165^ A recent retrospective study assessed the PK and pharmacodynamic parameters for 481 patients with disease caused by MAC.^122^ Peak serum concentrations within reference/normal ranges were only achieved for ethambutol, clarithromycin and azithromycin, in 52%, 44% and 65% of patients, respectively. In addition, pharmacodynamic targets for C_max_/MIC or AUC_0–24_/MIC were rarely achieved. However, these observations were not linked with clinical outcomes.

Another recent evaluation of the potential utility for TDM in 130 individuals without CF treated for MAC found no association between peak plasma/MIC ratios for clarithromycin, rifampin or ethambutol, and clinical outcomes.^171^ As previously observed, rifampin had a substantial impact on clarithromycin levels; those treated with both drugs had a median peak plasma clarithromycin concentration of 0.3 µg/mL, while those treated with rifabutin had a median peak plasma concentration of 1.8 µg/mL, and those with *M. abscessus* (n=60) treated without rifampin had a median peak plasma concentration of 3.8 µg/mL. In all, 97% of patients with MAC treated with daily therapy and 100% of patients on intermittent therapy reached the target of 2 µg/mL for clarithromycin. These experts concluded that TDM for treatment of MAC lung disease may not be beneficial (although the effects of dose optimisation on clinical outcomes were not evaluated).

To the best of our knowledge, there is only one case series, published over a decade ago, examining the potential role of TDM in CF. Ten patients with CF with mycobacterial disease (6 with MAC, 3 with *M. abscessus* and 1 with *M. tuberculosis*) had serum drug concentration measurements performed 2 and 4 h after ingestion.^164^ Monitoring serum levels at two time points helped distinguish between poor absorption and delayed absorption. Half of the patients had inadequate serum levels for one or more drugs, and one patient clinically improved following dose adjustments that achieved target serum levels. However, target concentrations were not achieved for several patients. Notably, this study did not compare outcomes in patients with and without TDM.

### Treatment: adjuvant therapy and surgery

In the context of infectious disease, adjuvants have been defined as ‘therapies that act by rendering the organism more susceptible to attack by antibiotics or the host immune system, by rendering the organism less virulent or killing it by other means’.^172^ A number of approaches have been proposed as candidates for adjuvant therapy in NTM infection in CF, including interferon γ (IFNγ; or agents that promote IFNγ release) and vitamin D. Drug delivery vehicles, such as liposomes, may be considered adjuvants. Liposomes have been studied as a mode of delivering amikacin for infection with *P. aeruginosa* in CF,^173^ and this approach is also being evaluated (clinicaltrials.gov/show/NCT01315236) for NTM.

#### Does IFNγ therapy improve treatment outcomes in individuals with CF who have NTM-PD?

***Recommendation 42*: The CF Foundation and the ECFS recommend against the use of IFNγ as adjuvant therapy for NTM-PD in individuals with CF.**

IFNγ plays a critical role in the host defence against NTM infection: (1) deficiencies in IFNγ signalling (caused by deleterious mutations^174^ or neutralising autoantibodies^175^) lead to (usually disseminated) NTM infection in individuals without CF; (2) inoculation of mice deficient in IFNγ or IFNγ receptors results in disseminated NTM infection;^176^ (3) addition of IFNγ to NTM-infected human macrophages in vitro enhances intracellular killing probably through autophagy stimulation.^43^

In non-CF individuals, adjuvant IFNγ therapy in NTM infection has been examined in several studies.^177^ ^178^ An uncontrolled trial of IFNγ was conducted in seven patients with presumed primary immunodeficiency (three with familial susceptibility to MAC and four with idiopathic CD4 lymphopaenia) who had disseminated NTM disease refractory to conventional antibiotic therapy.^177^ All the patients improved with the introduction of subcutaneous IFNγ two or three times per week.

In a randomised, placebo controlled trial, 32 patients with pulmonary NTM disease (30 with MAC) were randomised to receive either intramuscular IFNγ (1×10^6^ IU) or placebo once daily for 4 weeks and then three times weekly for 20 weeks^178^ in addition to daily oral azithromycin, ciprofloxacin, ethambutol and rifampin. The primary outcome (a composite end point of improvements in symptoms, radiology and microbiology) was achieved at 6 months by 72% (13/18) of patients in the IFNγ arm compared to 36% (5/14) receiving placebo (p=0.037). The greater response rate with IFNγ was sustained at 12 months after completion of treatment. However, the small study size, the use of composite end points and the lack of microbiological response after 6 months treatment mean that these data need to be interpreted with caution.

Furthermore three large trials (ClinicalTrials.gov Identifiers NCT00001318, NCT00111397 and NCT00043355) examining IFNγ therapy for pulmonary NTM disease remain unpublished or have been terminated (potentially due to lack of efficacy), again questioning the role of IFNγ adjuvant therapy.

#### Does vitamin D supplementation improve treatment outcomes in individuals with CF who have NTM-PD?

***Recommendation 43*: The CF Foundation and the ECFS recommend that vitamin D should be supplemented according to national CF care guidelines.**

Vitamin D is thought to play a critical role in host defence against mycobacteria. In vitro and ex vivo treatment with vitamin D of human macrophages infected with *M. tuberculosis* enhances intracellular killing (through stimulating antimicrobial peptide production^179^ and autophagy.^180^) Furthermore, several epidemiological studies have shown an association of vitamin D deficiency with reactivation of TB^181^ ^182^ and, recently, the presence of NTM-PD.^71^ However, interventional trials of vitamin D supplementation in patients with active pulmonary TB have had mixed results,^183^ and there are no trials of vitamin D as an adjuvant treatment for NTM disease.

#### Should surgery be considered in individuals with CF who have NTM-PD?

***Recommendation 44*: The CF Foundation and the ECFS recommend that lung resection should only be considered under extraordinary circumstances and in consultation with experts on the treatment of NTM and CF.**

Surgical resection has been used extensively in the management of pulmonary mycobacterial infection in order to excise localised infection, debulk severe disease, or excise cavities or damaged lung into which antibiotic penetration may be impaired. In no cases has surgery been used as a substitute for antibiotic therapy. There are no randomised trials of surgery for the treatment of pulmonary NTM disease in any patient group. While many publications report the use of lung resection (pneumonectomy, lobectomy or segmentectomy) with combination antibiotic therapy in NTM infection, most are case reports with no comparator group receiving only medical therapy thereby preventing objective assessment of the efficacy of surgery.

Nonetheless, three series of individuals without CF do contain some comparison data although the potential for selection bias of patients considered suitable for surgery makes interpretation difficult. The first series^136^ comprised of 65 patients, from South Korea, with pulmonary *M. abscessus* infection. Surgery was performed in 14 patients who failed to achieve sputum culture conversion, became culture positive again after a period of culture negativity or experienced disease-related complications such as haemoptysis. Of the eight patients who were sputum culture positive before surgery, seven became culture negative postoperatively (compared to culture conversion rates of 38/65 for the group as a whole). A second study, from the USA,^129^ reported outcomes for 69 patients with pulmonary *M. abscessus* infection all treated with combination antibiotics, 23 of whom underwent additional surgical resection. Indications for surgery included the presence of localised bronchiectasis, cavitary disease and haemoptysis. In the surgical group, significantly more patients (13/23) became persistently sputum culture negative compared to those in the medical treatment only group (13/46). A third study, also from the USA, described outcomes in 51 patients with macrolide-resistant MAC-PD.^126^ Individuals receiving both surgical resection and injectable aminoglycoside therapy had greater sputum conversion rates (11/14 patients) than those receiving neither treatment modality (2/37 patients).

A recent review of case series published over the last 40 years^184^ suggests that localised resection (lobectomy or segmentectomy) should be considered for severe, localised, unilateral NTM disease that has failed to respond to conventional antibiotic therapy. In the context of CF, however, localised NTM disease is extremely rare (or at least very difficult to identify), and the risks of thoracic surgery are high and therefore the potential benefits of surgical resection limited.

## Transplantation

### Should individuals with CF with current or previous NTM-positive cultures be referred for lung transplantation?

***Recommendation 45*: The CF Foundation and the ECFS recommend that all individuals with CF being considered for lung transplantation should be evaluated for NTM-PD.**

***Recommendation 46*: The CF Foundation and the ECFS recommend that the presence of current or previous respiratory tract samples positive for NTM should not preclude individuals being considered for lung transplantation.**

***Recommendation 47*: The CF Foundation and the ECFS recommend that individuals with CF who have NTM-PD and are being evaluated for transplantation should start treatment prior to transplant listing.**

***Recommendation 48*: The CF Foundation and the ECFS recommend that individuals with CF receiving NTM treatment with sequential negative cultures may be eligible for transplant listing.**

***Recommendation 49*: The CF Foundation and the ECFS recommend that individuals with CF who have completed treatment for NTM-PD with apparent eradication of the organism may be eligible for transplant listing.**

***Recommendation 50*: The CF Foundation and the ECFS recommend that the presence of persistent MABSC or MAC infection despite optimal therapy is not an absolute contraindication to lung transplant referral.**

The International Society for Heart and Lung Transplantation (ISHLT) International Guidelines lists ‘colonisation with highly resistant or virulent mycobacteria’ as a relative contra-indication for selection as a lung transplant candidate.^185^ There is, however, limited published information on transplant outcomes for individuals with previous or concurrent NTM infection, with very few reports (usually from single centres) specifically examining CF cohorts.^186^ ^187^

The risk of NTM infection post-transplantation is not well defined. A study of 201 CF and non-CF transplant recipients^188^ suggested that postoperative NTM acquisition was associated with increased mortality (HR 2.61), independent of bronchiolitis obliterans syndrome. However, these data should be interpreted keeping the following in mind: very little data were available on the presence of pulmonary NTM pretransplant; the vast majority of patients did not have CF or even bronchiectasis; and non-NTM-related causes were major contributors to death in fatal cases. In contrast, a recent study of CF and non-CF transplant recipients^189^ reported that 53 of 237 individuals (22.4%) acquired NTM-positive cultures postoperatively (70% MAC, 10% MABSC), of whom two fulfilled ATS/IDSA criteria for NTM-PD. Although overall mortality was not affected by NTM acquisition, four patients developed persistent surgical site infection (three with *M. abscessus*), of whom one died of disseminated NTM infection. The potential for *M. abscessus* to cause significant postoperative complications is supported by a review of outcomes from 31 transplant centres^190^ indicating frequent soft tissue and surgical site infections, and two deaths, attributable to *M. abscessus* infection.

The largest CF-specific case series comes from the University of North Carolina Chapel Hill experience between 1990 and 2003.^29^ One hundred and forty-six patients with CF underwent lung transplantation and 31 listed for transplantation. Of those individuals referred, 19.7% were NTM culture positive pretransplant. Rates of NTM following lung transplantation were 3.4%. Pretransplant infection with *M. abscessus* was recognised as a significant risk factor for recurrence of NTM post-transplantation. Although there was no effect on mortality, post-transplant NTM infection caused significant morbidity as patients developed *M. abscessus-*associated skin and soft tissue infection or pulmonary disease caused by MAC and other NTM species. There are several published case series of successful outcomes for individuals with CF who have culture positive *M. abscessus* infection at the time of transplantation.^186^ However, NTM-related complications in this group may be more frequent, and include persistent soft tissue or wound infections,^186^ empyema and disseminated NTM infection.^164^ ^191^ Although a small series, the University of North Carolina (UNC) report suggests no effect of the presence of pretransplant *M. abscessus* positive cultures on post-transplant mortality.^187^

The Consensus statements Committee concluded that all individuals with CF should be evaluated for NTM disease prior to referral for lung transplantation, given the very high reported rates of NTM culture positivity for this group, and the fact that untreated NTM infection may represent an increased (and potentially modifiable) postoperative risk. Consequently, if NTM-PD is diagnosed, treatment should be started prior to transplant listing.

## Conclusion

The management of individuals with CF infected with NTM is extremely challenging. The limited amounts of published research and clinical trial data provide inadequate evidence to base management decisions on how best to screen, diagnose, detect and treat NTM-PD. As a response to this urgent clinical need, the CF Foundation and the ECFS formed a committee of clinicians, scientists and infectious disease experts to develop recommendations to guide and assist clinicians in the management of NTM-PD in individuals with CF. The committee believes these recommendations should serve as a benchmark for current medical care while providing a framework to inform the development of clinical, translation and basic research studies to generate robust evidence on which to base future iterations of these management guidelines, leading to better outcomes for individuals with CF infected with NTM.

## Supplementary Material

Web NTM-recommendations

Web executive-summary
