# Supplementary material for: US Cystic Fibrosis Foundation and European Cystic Fibrosis Society consensus recommendations for the management of non-tuberculous mycobacteria in individuals with cystic fibrosis
Source: Thorax. 2015 Dec 11;71(Suppl 1):i1–i22. doi: 10.1136/thoraxjnl-2015-207360 (PMC4717371; doi:10.1136/thoraxjnl-2015-207360)
Supplement: Web NTM-recommendations [file thoraxjnl-2015-207360-s1.pdf]

Figure 1. CF Foundatation and European CF Society Recommendations on NTM management in CF

Screening	***1.*** The potential for cross-infection of NTM (particularly *M. abscessus* complex) between individuals with CF should be minimised by following national infection control guidelines.
	2. Cultures for NTM be performed annually in spontaneously expectorating individuals with a stable clinical course.
	3. In the absence of clinical features suggestive of NTM pulmonary disease, individuals who are not capable of spontaneously producing sputum do not require screening cultures for NTM.
	4. Culture and smears for acid fast bacilli from sputum should be used for NTM screening.
	5. Oro-pharyngeal swabs should not be used for NTM screening.
Microbiology	***6.*** Cultures and smears for acid fast bacilli (AFB) from sputum, induced sputum, bronchial washings or broncho-alveolar lavage samples can be used to evaluate individuals with CF suspected to have NTM pulmonary disease.
	7. Transbronchial biopsies should not be routinely used to detect NTM in individuals with CF suspected to have NTM pulmonary disease.
	8. Oro-pharyngeal swabs should not be used to perform diagnostic smears and cultures in individuals with CF suspected to have NTM pulmonary disease.
	9. Respiratory tract samples should be cultured using both solid and liquid media.
	10. The incubation duration for NTM cultures should be for a minimum of 6 weeks.
	11. An NTM culture should be processed within 24 hours of collection to optimize the detection of NTM in respiratory samples. If a delay in processing is anticipated, refrigeration of samples is advised.
	12. Respiratory tract samples should be decontaminated using the standard N-Acetyl L-cysteine NALC (0.5%) – NaOH (2%) method.
	13. If a sample remains contaminated with gram-negative bacteria after standard NALC-NaOH decontamination, it should be further treated with either 5% oxalic acid or 1% chlorhexidine.
	14. Non-culture based methods should not be used for detecting NTM in respiratory tract samples.
	15. All NTM isolates from individuals with CF should undergo molecular identification.
	16. All NTM isolates from individuals with CF should be identified to the species level, except for *M. intracellulare, M. avium and M. chimaera*, where identification can be limited to *M. avium* complex (MAC), and *M. abscessus* complex, which should be sub-speciated.
	17. For *M. avium* complex, clarithromycin susceptibility testing should be performed on an isolate recovered prior to initiation of treatment. Clarithromycin susceptibility testing should also be performed on subsequent isolates if the patient a) fails to culture convert after six months of NTM treatment; b) recultures *M. avium* complex after initial culture conversion while on NTM treatment; or c) recultures *M. avium* complex after completion of NTM treatment.
	18. For *M. abscessus* complex, susceptibility testing should include at least clarithromycin, cefoxitin and amikacin (and preferably also tigecycline, imipenem, minocycline, moxifloxacin and linezolid).
	19. Drug susceptibility testing should be performed in accordance with CLSI guidelines.
Diagnosis	***20.*** ATS/IDSA criteria for the diagnosis of NTM pulmonary disease should be used in individuals with CF
	21. Other CF pathogens and co-morbidities should be considered as potential contributors to a patient's symptoms and radiological features when determining the clinical significance of NTM positive cultures.
	22. NTM treatment should be considered for individuals with CF who have ATS/IDSA defined NTM pulmonary disease.
	23. Individuals receiving azithromycin as part of their CF medical regimen who have a positive NTM culture should not continue azithromycin treatment while evaluation for NTM disease is underway as azithromycin monotherapy may lead to resistance. A macrolide agent may be included in a multi-drug treatment regimen if criteria are met for NTM disease.
Treatment	***24.*** Treatment of *M. abscessus* complex pulmonary disease should involve an intensive phase followed by a continuation phase.
	25. The intensive phase should include a daily oral macrolide (preferably azithromycin) in conjunction with 3-12 weeks of intravenous amikacin and one or more of the following: intravenous tigecycline, imipenem or cefoxitin, guided but not dictated by drug susceptibility testing. The duration of intensive phase therapy should be determined by the severity of infection, the response to treatment and the tolerability of the regimen.
	26. The continuation phase should include a daily oral macrolide (preferably azithromycin) and inhaled amikacin, in conjunction with 2-3 of the following additional oral antibiotics: minocycline, clofazimine, moxifloxacin and linezolid, guided but not dictated by drug susceptibility testing.
	27. Individuals with *M. abscessus* complex pulmonary disease should be managed in collaboration with experts in the treatment of NTM and CF as drug intolerance and drug-related toxicity occur frequently and changes in antibiotic therapy are often required.
	28. Monotherapy with a macrolide or other antimicrobial should never be used in the treatment of *M. abscessus* complex pulmonary disease.
	29. The same antibiotic regimen should be used for treatment of all species within the *M. avium* complex.
	30. Clarithromycin-sensitive *M. avium* complex pulmonary disease should be treated with a daily oral antibiotic regimen containing a macrolide (preferably azithromycin), rifampin and ethambutol.
	31. Intermittent (three-times-per week) oral antibiotic therapy should not be used to treat *M. avium* complex pulmonary disease.
	32. Monotherapy with a macrolide or other antimicrobial agent should never be used in the treatment of *M. avium* complex pulmonary disease.
	33. An initial course of intravenous amikacin should be considered for the treatment of *M. avium* complex pulmonary disease in the presence of one or more of the following: i)AFB smear positive respiratory tract samples; ii) Radiological evidence of lung cavitation or severe infection; iii) Systemic signs of illness.
	34. Clarithromycin-resistant *M. avium* complex pulmonary disease should be managed in collaboration with experts in the treatment of NTM and CF.
	35. Individuals with CF receiving NTM treatment should have expectorated or induced sputum samples sent for NTM culture every 4-8 weeks throughout the entire course of treatment to assess the microbiological response.
	36. A schedule for detecting drug toxicity (including hearing loss, visual loss, renal impairment and liver function test abnormalities) should be set in place at the time of NTM treatment initiation and implemented throughout treatment based on the specific drugs prescribed.
	37. An HRCT scan of the lungs should be performed shortly before starting NTM treatment and at the end of NTM treatment to assess the radiological response.
	38. NTM antibiotic therapy should be prescribed for 12 months beyond culture conversion (defined as three consecutive negative cultures, with the time of conversion being the date of the first of the three negative cultures) as long as no positive cultures are obtained during this 12 months.
	39. Individuals who fail to culture convert despite optimal NTM therapy may benefit from long term suppressive antibiotic treatment.
	40. When amikacin is given intravenously or when streptomycin is given intravenously or intramuscularly, serum levels should be monitored and dosing adjusted to minimize ototoxicity and nephrotoxicity.
	41. Serum levels of other anti-mycobacterial drugs should not be routinely obtained. However, absorption of oral medications is often reduced in CF. Therefore use of therapeutic drug monitoring should be considered for individuals failing to improve despite taking recommended drug regimens or for those on concomitant medications with significant interactions with NTM drugs.
	42. Interferon gamma should not be used as adjuvant therapy for NTM pulmonary disease in individuals with CF.
	43. Vitamin D should be supplemented according to national CF care guidelines.
	44. Lung resection should only be considered in extraordinary circumstances and in consultation with experts in the treatment of NTM and CF.
Transplantation	***45.*** All individuals with CF being considered for lung transplantation should be evaluated for NTM pulmonary disease.
	46. The presence of current or previous respiratory tract samples positive for NTM should not preclude individuals being considered for lung transplantation.
	47. Individuals with CF who have NTM pulmonary disease and are being evaluated for transplantation should commence treatment prior to transplant listing.
	48. Individuals with CF receiving NTM treatment with sequential negative cultures may be eligible for transplant listing.
	49. Individuals with CF who have completed treatment for NTM pulmonary disease with apparent eradication of the organism may be eligible for transplant listing.
	50. The presence of persistent *M. abscessus* complex or *M. avium* complex infection despite optimal therapy is not an absolute contraindication to lung transplant referral.
