# Supplementary material for: US Cystic Fibrosis Foundation and European Cystic Fibrosis Society consensus recommendations for the management of non-tuberculous mycobacteria in individuals with cystic fibrosis
Source: Thorax. 2015 Dec 11;71(Suppl 1):i1–i22. doi: 10.1136/thoraxjnl-2015-207360 (PMC4717371; doi:10.1136/thoraxjnl-2015-207360)
Supplement: Web executive-summary [file thoraxjnl-2015-207360-s2.pdf]

Cystic Fibrosis Foundation and European Cystic Fibrosis Society Consensus Recommendations for the Management of Nontuberculous Mycobacteria in Individuals with Cystic Fibrosis –EXECUTIVE SUMMARY

R. Andres Floto[1,2], Kenneth N. Olivier[3], Lisa Saiman[4], Charles L. Daley[5], Jean-Louis Herrmann[6,7], Jerry A. Nick[8], Peadar G Noone[9], Diana Bilton[10], Paul Corris[11], Ronald L. Gibson[12], Sarah E. Hempstead[13], Karsten Koetz[14], Kathryn A. Sabadosa[13], Isabelle Sermet-Gaudelus[14], Alan R Smyth[16], Jakko van Ingen[17], Richard J. Wallace[18], Kevin L. Winthrop[19], Bruce C. Marshall[20] & Charles S. Haworth[2]

[1]Cambridge Institute for Medical Research, University of Cambridge, UK

[2]Cambridge Centre for Lung Infection Papworth Hospital, Cambridge, UK

[3]Cardiovascular and Pulmonary Branch, National Heart, Lung, and Blood Institute, NIH, Bethesda, MD, USA

[4]Department of Pediatrics, Columbia University Medical Center, Pediatric Infectious Diseases, New York, NY USA

[5]Division of Mycobacterial and Respiratory Infections & [8]Department of Medicine, National Jewish Health, Denver, CO USA

[6] INSERM U1173, UFR Simone Veil, Versailles-Saint-Quentin University, 78180 Saint-Quentin en Yvelines, France

[7] AP-HP, Service de Microbiologie, Hôpital Raymond Poincaré, 92380 Garches, France

[9]The University of North Carolina at Chapel Hill, NC USA

[10]Department of Respiratory Medicine, Royal Brompton Hospital, London, UK

[11]Department of Respiratory Medicine Freeman Hospital High Heaton Newcastle, UK

[12]Department of Pediatrics University of Washington School of Medicine, Seattle, WA

[13]The Dartmouth Institute for Health Policy and Clinical Practice, Geisel School of Medicine at Dartmouth, Lebanon, NH

[14]Department of Pediatrics, Sahlgrenska University Hospital, Gothenburg, Sweden

[15]Université René Descartes, Service de Pneumo-Pédiatrie , Hôpital Necker-Enfants Malades, Paris, France

[16]Division of Child Health, Obstetrics & Gynaecology, University of Nottingham, UK

[17]Department of Medical Microbiology, Radboud University Medical Center, Nijmegen, the Netherlands

[18]Dept. of Microbiology, University of Texas Health Science Center, Tyler TX, USA

[19]Oregon Health and Sciences University, Portland, OH, USA

[20]Cystic Fibrosis Foundation, Bethesda, MD, USA

Corresponding Author:
R. Andres Floto, Cambridge Institute for Medical Research, University of Cambridge, Cambridge Biomedical Campus, Hills Road, Cambridge, CB2 0XY UK Email: arf27@cam.ac.uk. Phone: +44 1223 768801. Fax: +44 1223 762640

Authors contribution:
All authors contributed to the expert committee on guidelines development (Co-chairs: RAF, CSH; Steering committee: RAF, CSH, BCM, KNO, LS, KAS, SEH; Committee subgroups: Epidemiology and Risk Factors (KNO (lead), IS-G, KLW): Screening (JAN (lead), RLG, KK; Microbiology (J-LH (lead), RJW, JvI, RAF); Treatment (CLD (lead), DB. LS, ARS, CSH); and Transplantation (PN (lead), PC).

Sources of Support: Cystic Fibrosis Foundation; European Cystic Fibrosis Society, The Wellcome Trust & Cambridge NIHR BRC (RAF); Intramural program of the National Heart, Lung, and Blood Institute, NIH (KNO); Vaincre La Mucoviscidose (VLMIC1014 and RF20120600689) and the Région Ile-de-France Domaine d'Intérêt Majeur Maladies Infectieuses et Emergentes (JLH); CF Foundation Clinical Research Award (NICK13A0) (JAN); Imperial College London NIHR Respiratory BRU (DB);.

Short Running Title:
Management guidelines for NTM infection in Cystic Fibrosis

Descriptor number:
11.5 (Nontuberculous mycobacteria
MeSH terms: cystic fibrosis; Nontuberculous mycobacteria

ABSTRACT

Nontuberculous mycobacteria (NTM) are ubiquitous environmental organisms that can cause chronic pulmonary infection, particularly in individuals with pre-existing inflammatory lung disease such as cystic fibrosis (CF). Pulmonary disease caused by NTM has emerged as a major threat to the health of individuals with CF but remains difficult to diagnose and problematic to treat. In response to this challenge, the U.S. Cystic Fibrosis Foundation (CFF) and the European Cystic Fibrosis Society (ECFS) convened a panel of 19 experts to develop consensus recommendations for the screening, investigation, diagnosis and management of NTM pulmonary disease in individuals with CF. PICO methodology and systematic literature reviews were employed to inform draft recommendations which were then modified to achieve consensus and subsequently circulated for public consultation within the US and European CF communities. We have thus generated a series of pragmatic, evidence-based recommendations as an initial step in optimising management for this challenging condition.

BACKGROUND:

Nontuberculous mycobacteria (NTM) are increasingly being isolated from the sputum of adults and children with cystic fibrosis (CF) both in North America and Europe. Estimates of the prevalence of NTM in the CF population have ranged from 1.3% in the earliest study reported in 1984(1) to 32.7% in a review of CF patients over age 40 in Colorado(2).

The NTM species most commonly identified in individuals with CF from North America and Europe are the slow growing *Mycobacterium avium* complex (MAC) (including *M. avium*, *M. intracellulare* and *M. chimaera*) which can be found in up to 72% of NTM positive sputum cultures and the rapid growing *M. abscessus* complex (MABSC), (comprising the subspecies *M. abscessus subsp. abscessus* (*M. a. abscessus)*, *M. a. bolletii* and *M. a. massiliense* (the latter currently classified as part of *M. a. bolletii*) which in many centres has now become the most common NTM isolated from individuals with CF.

There has been a rise in the prevalence of NTM positive cultures in respiratory samples from individuals with CF over the last three decades which probably reflects a true increase in the frequency of NTM infection. A number of CF studies (for example Renna et al(3)) show year on year increases in NTM positive cultures with no change in surveillance intensity or culture methodology.

Possible reasons for increased NTM positive cultures in individuals with CF include: increases in environmental exposure to NTM through more permissive temperature settings of home water heaters and more contact with shower aerosols, increased antibiotic usage creating more NTM permissive lung niches, greater chronic use of medications which might impair host immunity to NTM (3) and/or spread of NTM through person-to-person transmission (4).

NTM can cause progressive inflammatory lung damage, a condition termed ‘NTM pulmonary disease’ (NTM-PD) , which is defined by the presence of specific microbiological, clinical and radiological features (5). However, it has become clear that NTM can also transiently, intermittently, or permanently reside within the lungs of CF individuals without causing NTM-PD, thus representing asymptomatic infection and creating considerable difficulties in deciding how best to screen for and diagnose NTM.

Further challenges exist in knowing how best to identify NTM in respiratory samples, when and how to initiate treatment for NTM-PD and how NTM may impact individuals under consideration for lung transplantation. As a consequence, the CFF and ECFS sought to generate a consensus recommendations document to support and standardize the management of NTM infection in children and adults with CF, permitting prospective evaluation of current best practice and forming a foundation for future research programs.

METHODS

The Cystic Fibrosis Foundation and the European Cystic Fibrosis Society (ECFS) invited experts to participate in the statement development process. The 19 member committee consisted of professionals with expertise in CF and NTM and included adult and pediatric CF physicians, lung transplant physicians, microbiologists, infectious disease specialists, and a parent of an individual with CF. The committee convened in May 2012 and divided into 5 sub-groups, each responsible for a specific topic: Epidemiology and Risk Factors, Screening, Microbiology, Treatment, and Transplantation. Each sub-group developed topic specific questions using the PICO format (Population, Intervention, Comparison, Outcome). Questions were reviewed and approved by the entire committee before systematic literature searches were conducted.

The members of each sub-group used the PICO questions to guide literature searches in PubMed. Searches were limited to English language and the period 1984 to 2013. Sub-group members also searched for topic relevant guidelines through searches of the ATS website, the IDSA website, the Clinical Laboratory Standards Institute website, and the United Kingdom (UK) CF Trust website.

After reviewing relevant literature and existing guidelines, sub-group members drafted recommendation statements. In October 2012, a second meeting was convened and sub-groups finalized draft recommendation statements. The committee also voted to set 80% agreement of all 19 members as the threshold for acceptance of a

recommendation statement.

Each sub-group submitted final draft questions for entry into an electronic survey tool (Survey Monkey) for the purposes of anonymous voting and comment by all members. A project coordinator administered the survey and committee members were asked to rate each statement on a scale of 0, completely disagree, to 9, completely agree, with 80% or between 7 and 9 being considered 'good' agreement. Space for entering free text was also provided after each statement to allow members to cite literature in support of their opinions or suggested revisions. All committee members were required to vote on each statement regardless of their role or expertise. Multiple rounds of voting and revisions to the statements were conducted and for each round committee members were requested to complete their voting within 3 weeks. The committee chairs reviewed the results from each round and updated the statements based on comments entered by respondents for subsequent rounds.

A draft of the recommendations was presented at the 2013 North American Cystic Fibrosis Conference and the European Cystic Fibrosis Society Meeting. Additionally the committee solicited feedback from the CF communities in the U.S. and Europe, which included physicians, nurses, physical and respiratory therapists, parents and individuals with CF. Comments collected from this process were considered by the committee in the development of the final recommendation statements.

RESULTS

Three rounds of voting were conducted to achieve 80% consensus for each statement. Fifty-three statements were included in the first round of voting and 50 statements in the second and third rounds. Final statements are shown in Figure 1.

DISCUSSION

The management of individuals with CF infected with NTM is extremely challenging. The limited amounts of published research and clinical trial data provide inadequate evidence to base management decisions on how best to screen, diagnose, detect and treat NTM-pulmonary disease. As a response to this urgent clinical need, the CF Foundation and ECFS formed a committee of clinicians, scientists and infectious disease experts to develop recommendations to guide and assist clinicians in the management of NTM-PD in individuals with CF. The committee believe these recommendations should serve as a benchmark for current medical care while providing a framework to inform the development of clinical, translation and basic research studies to generate robust evidence to base future iterations of these management guidelines leading to better outcomes for individuals with CF infected with NTM.

REFERENCES

1. Smith MJ, Efthimiou J, Hodson ME, Batten JC. Mycobacterial isolations in young adults with cystic fibrosis. *Thorax* 1984;39(5):369-375.

2. Rodman DM, Polis JM, Heltshe SL, Sontag MK, Chacon C, Rodman RV, Brayshaw SJ, Huitt GA, Iseman MD, Saavedra MT, et al. Late diagnosis defines a unique population of long-term survivors of cystic fibrosis. *Am J Respir Crit Care Med* 2005;171(6):621-626.

3. Renna M, Schaffner C, Brown K, Shang S, Tamayo MH, Hegyi K, Grimsey NJ, Cusens D, Coulter S, Cooper J, et al. Azithromycin blocks autophagy and may predispose cystic fibrosis patients to mycobacterial infection. *J Clin Invest* 2011;121(9):3554-3563.

4. Bryant JM, Grogono DM, Greaves D, Foweraker J, Roddick I, Inns T, Reacher M, Haworth CS, Curran MD, Harris SR, et al. Whole-genome sequencing to identify transmission of mycobacterium abscessus between patients with cystic fibrosis: A retrospective cohort study. *Lancet* 2013;381(9877):1551-1560.

5. Griffith DE, Aksamit T, Brown-Elliott BA, Catanzaro A, Daley C, Gordin F, Holland SM, Horsburgh R, Huitt G, Iademarco MF, et al. An official ats/idsa statement: Diagnosis, treatment, and prevention of nontuberculous mycobacterial diseases. *Am J Respir Crit Care Med* 2007;175(4):367-416.
